# PP1A Modulates the Efficacy of Lenvatinib Plus ICIs Therapy by Inhibiting Ferroptosis in Hepatocellular Carcinoma

**DOI:** 10.1002/advs.202501730

**Published:** 2025-05-08

**Authors:** Jitong Zhou, Meng Gao, Shikun Zhang, Wing‐Wa Guo, Wenzhi He, Minghe Zhang, Xi Chen, Cairang Dongzhi, Xiaomian Li, Yufeng Yuan, Weijie Ma

**Affiliations:** ^1^ Department of Hepatobiliary and Pancreatic Surgery Zhongnan Hospital of Wuhan University Wuhan 430071 P. R. China; ^2^ Clinical Medicine Research Center for Minimally Invasive Procedure of Hepatobiliary & Pancreatic Diseases of Hubei Province Wuhan 430071 P. R. China; ^3^ TaiKang Center for Life and Medical Sciences Wuhan University Wuhan 430071 P. R. China

**Keywords:** combination therapy, ferroptosis, immune checkpoint inhibitors, lenvatinib, PD‐L1, PP1A

## Abstract

Advanced hepatocellular carcinoma (HCC) is characterized by poor prognosis, primarily due to limited therapeutic options and resistance to treatment. Although the combination of tyrosine kinase inhibitors (TKIs) and immune checkpoint inhibitors (ICIs) has shown promising potential, the underlying mechanisms remain inadequately understood. Here, serine/threonine‐specific protein phosphatase (PP1A) is upregulated in Lenvatinib‐resistant HCC cells and correlates with poor prognosis. Functional experiments revealed that PP1A promotes HCC progression both in vitro and in vivo. Transcriptomic analysis and ferroptosis metabolite profiling (e.g., ROS, Fe^2^⁺, lipid‐ROS, and GSH) demonstrated that PP1A inhibits Lenvatinib‐induced ferroptosis by dephosphorylating Keap1 at site 104. This disruption of the Keap1‐Nrf2 interaction enhances the transcription of ferroptosis‐related markers and immune checkpoint PD‐L1. Notably, single‐cell sequencing and co‐culture experiments revealed that PP1A knockdown alleviates T cell exhaustion and immune evasion, thereby improving antitumor immunity. In vivo experiments further demonstrated that PP1A knockdown significantly enhances the efficacy of Lenvatinib‐ICIs combination therapy. Overall, our findings highlight PP1A as a critical regulator of ferroptosis and antitumor immunity, suggesting its potential as a predictive biomarker and therapeutic target for improving outcomes in advanced HCC.

## Graphical abstract

PP1A dephosphorylates Keap1 at the S104 site, stabilizing and promoting the nuclear translocation of Nrf2, which upregulates the expression of antioxidant genes and PD‐L1. This leads to suppressed ferroptosis and enhanced immune evasion, thereby improving the efficacy of TKI and ICI combination therapy.

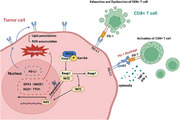

## 1 Introduction

Hepatocellular carcinoma (HCC) is the third leading cause of cancer‐related death worldwide.^[^ ^1^ ^]^ Owing to the late symptom onset, more than 50% of HCC patients are diagnosed at advanced stages.^[^ ^2^ ^]^ Early‐stage HCC can often be resected, whereas systemic treatment is typically needed for advanced HCC.^[^ ^3^ ^]^ Two tyrosine kinase inhibitors (TKIs), Sorafenib and Lenvatinib, are approved as first‐line therapeutic drugs.^[^ ^4^ ^]^ Lenvatinib, serving as an alternative to Sorafenib, has a better objective response, disease control rate, and manageable tolerability profile for HCC patients.^[^ ^5^ ^]^ Despite the initial response, most patients eventually acquire Lenvatinib resistance and disease progression, limiting its efficacy and clinical application as a single agent.^[^ ^6^ ^]^ Therefore, exploring the underlying mechanism of Lenvatinib resistance is crucial for improving HCC prognosis. Recently, immunotherapy has successfully transformed the treatment landscape of HCC.^[^ ^7^ ^]^ Immune checkpoint inhibitors (ICIs), especially those targeting the PD‐1/PD‐L1 axis, have shown substantial antitumor effects in some HCC patients.^[^ ^8^ ^]^ However, more than 30% of advanced HCC tumors exhibit intrinsic resistance to PD‐1 or PD‐L1 inhibitors.^[^ ^9^ ^]^ Increasing evidence suggests that the clinical response to anti‐PD‐1/PD‐L1 therapy is highly dependent on PD‐L1 expression on tumor cells and T lymphocyte infiltration.^[^ ^10^ ^]^ A comprehensive understanding of the mechanisms that regulate PD‐L1 expression may facilitate the development of novel therapeutic strategies to improve the efficacy of PD‐1/PD‐L1 blockade.

Ferroptosis is a recently identified form of regulated cell death (RCD) characterized by the iron‐dependent accumulation of lethal lipid reactive oxygen species (ROS).^[^ ^11^ ^]^ We previously found that both Sorafenib and Lenvatinib can induce ferroptosis in HCC.^[^ ^12^ ^]^ Recent research also demonstrated that the induction of ferroptosis reversed TKIs resistance in cancer cells.^[^ ^13^ ^]^ These findings suggest that targeting ferroptosis may increase the efficacy of cancer treatment. Moreover, several studies have suggested that inducing ferroptosis in cancer cells can promote the infiltration of immune cells in the tumor microenvironment (TME) and enhance anti‐tumor immunity.^[^ ^14^ ^]^ Therefore, a novel strategy that increases the vulnerability of tumor cells to ferroptosis might improve the therapeutic efficacy and offer more possibilities for combination therapy in HCC.

By sequencing Lenvatinib‐resistant HCC cell lines, we identify PP1A as a hallmark molecule. serine/threonine‐specific protein phosphatase (PP1A) has emerged as a key tumor‐specific target because of its role in dephosphorylation.^[^ ^15^ ^]^ It has been reported that PP1A expression is increased in various types of cancer and is associated with tumor progression and a poor prognosis.^[^ ^15^, ^16^ ^]^ However, the role of PP1A in the regulation of ferroptosis and antitumor immunity remains unclear. Our study aimed to systematically investigate the role of PP1A in HCC, particularly in Lenvatinib resistance, and to explore the relationship between PP1A and the Keap1‐Nrf2 signaling pathway, and explore its impact on antitumor immune response. Additionally, we systematically evaluated and compared the role of PP1A in the response to monotherapy and combination therapy with Lenvatinib and ICIs in mouse models. Our research not only contributes to understanding the biological function of PP1A as a potential therapeutic target but also may provide theoretical support for its use as a new molecular target for the development of combined treatment strategies involving Lenvatinib and ICIs in HCC.

## 2 Results

### 2.1 PP1A Upregulation in HCC Predicts Poor Prognosis and Drives Resistance to Lenvatinib

**Figure 1:**
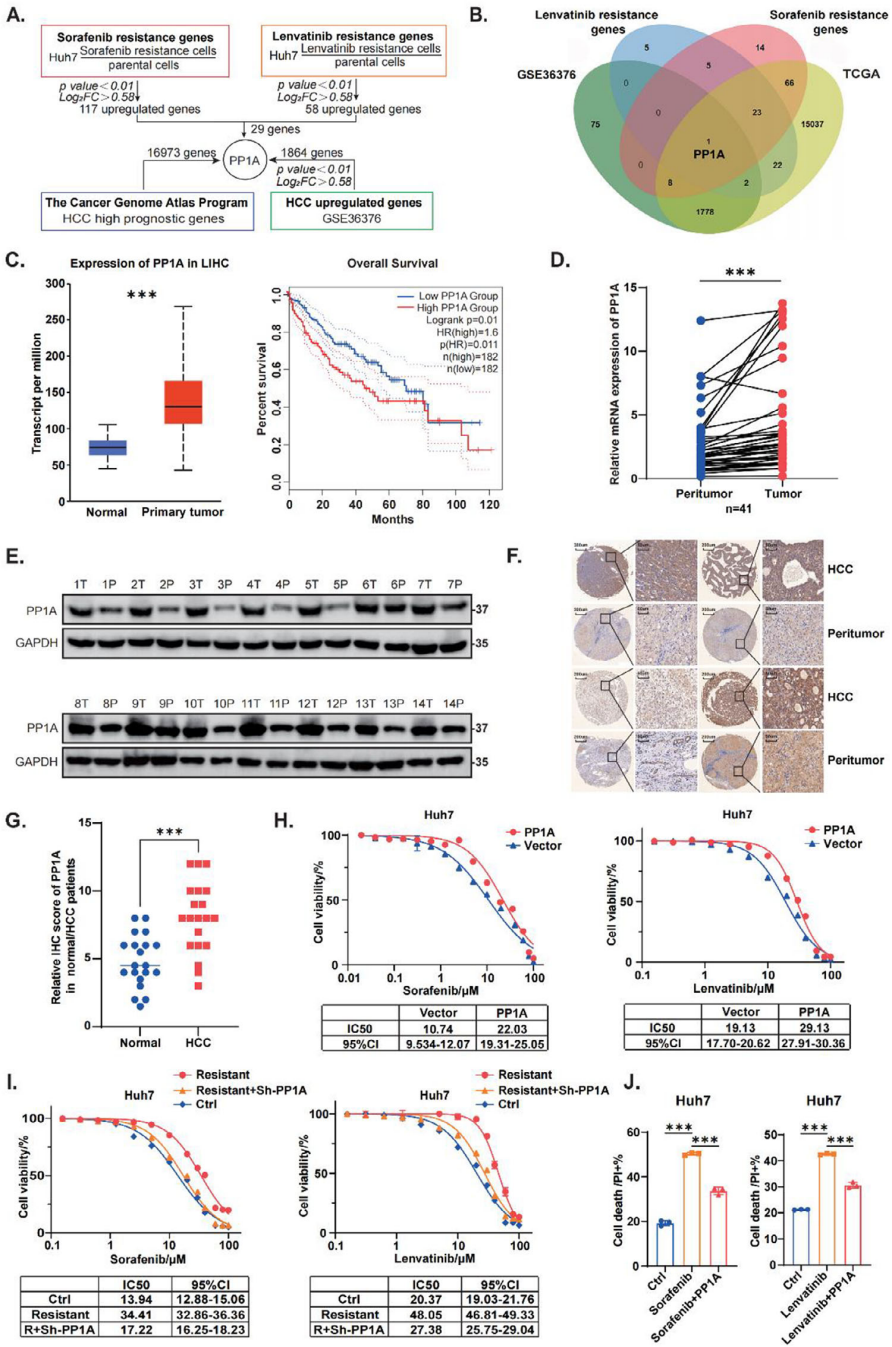
PP1A upregulation in HCC predicts poor prognosis and drives resistance to Lenvatinib. A‐B) Sorafenib/Lenvatinib‐resistant cell lines were established by exposing cells to increasing concentrations (IR) of Sorafenib/Lenvatinib. A total of 29 genes associated with resistance to both Sorafenib and Lenvatinib were identified by comparing Sorafenib/Lenvatinib‐resistant Huh7 cell lines with their parental cell lines. Venn diagram analysis of the GEOx and TCGA databases led to the identification of PP1A as a candidate gene. C) PP1A expression in tumor tissues from the LIHC‐TCGA dataset, and its association with prognosis. D) The expression of PP1A mRNA in paired HCC tissues and adjacent non‐tumor tissues (n = 41) was analyzed by qRT‐PCR. E) The expression of PP1A protein in paired HCC tissues and adjacent non‐tumor tissues (n = 14) was analyzed by Western blot. F) Immunohistochemical staining (IHC) was performed to evaluate PP1A expression in normal liver tissues and HCC tissues. G) Statistical analysis of the IHC staining scores (n = 20) was conducted to compare PP1A expression levels between HCC tissues and adjacent non‐tumor tissues. H) The Sorafenib/Lenvatinib resistance curves were plotted for control and PP1A‐overexpressing Huh7 cell lines, and changes in IC50 values were compared (n = 3). I) The Sorafenib/Lenvatinib resistance curves were plotted for the control, Sorafenib/Lenvatinib‐resistant cells, and PP1A‐knockdown resistant cell lines, and changes in IC50 values were compared (n = 3). J) Cell death was assessed and compared among the control, Sorafenib/Lenvatinib‐treated, and PP1A‐overexpressing Sorafenib/Lenvatinib‐treated groups (n = 3). Except for Figure 1D,G, which used paired *t*‐test, and Figure 1H,I, which used nonlinear regression, unpaired Student's t‐test or one‐way ANOVA was used to analyze the data. ^*^ *p* < 0.05, ^**^ *p* < 0.01, ^***^ *p* < 0.001. n.s, not significant. The data are expressed as the mean ± SD of three independent experiments. T, tumor tissues; P, para‐tumor normal tissues.

Recent studies have demonstrated that inhibition of ferroptosis plays a significant role in Lenvatinib resistance in HCC.^[^ ^12b^ ^]^ Therefore, we established HCC cell models resistant to Lenvatinib and Sorafenib and identified 29 commonly upregulated genes, potentially involved in ferroptosis inhibition. Moreover, we screened 16,973 genes from The Cancer Genome Atlas (TCGA) and Genotype‐Tissue Expression (GTEx) database (GSE36376), ultimately identifying PP1A as a key gene in HCC (**Figure** **1**A,B). Analysis of the Human Protein Atlas (HPA) database revealed high expression of PP1A in various tumor cells and its positive impact on prognosis (Figure S1A,B, Supporting Information). Moreover, analysis of GEO microarray datasets (GSE223201 and GSE213615) further confirmed high expression of PP1A in Sorafenib‐ and Lenvatinib‐resistant HCC (Figure S1C,D, Supporting Information). We observed elevated PP1A protein and mRNA levels in Lenvatinib‐resistant cells compared to parental cells (Figure S1E,F, Supporting Information). A dual‐luciferase reporter assay showed increased PP1A promoter activity in resistant cell lines (Figure S1G, Supporting Information), indicating that Lenvatinib may raise PP1A levels by activating its transcription. We also conducted a screening to identify transcription factors that may play a functional role (Figure S1H, Supporting Information). Previous studies have suggested a relationship between PP1A and tumor development. We detected elevated PP1A expression in HCC in the UALCAN cancer database, and we found that high PP1A expression was associated with a poor prognosis in the TCGA database (Figure 1C). Elevated PP1A expression in liver cancer tissues was also validated in the HPA database (Figure S1I, Supporting Information). We further validated the elevated expression of PP1A in HCC clinical specimens via quantitative real‐time PCR (qRT‐PCR) (Figure 1D) and Western blot (Figure 1E). Immunohistochemistry (IHC) revealed higher PP1A expression in tumor tissues than in normal liver tissues (Figure 1F,G). To assess the role of PP1A in Sorafenib or Lenvatinib resistance in HCC, we overexpressed PP1A in Huh7 cells. The stable overexpression cell lines showed higher IC50 values for both Sorafenib and Lenvatinib (Figure 1H). Knockdown of PP1A in resistant cells reversed resistance to Sorafenib and Lenvatinib (Figure 1I). Apoptosis assays revealed that overexpression of PP1A reduced cell death following Sorafenib or Lenvatinib treatment (Figure 1J). In conclusion, PP1A is found to be upregulated in HCC, associated with poor prognosis, and identified as a key regulator of Lenvatinib resistance.

### 2.2 PP1A Promotes the Proliferation, Invasion, and Metastasis of HCC Cells In Vitro and In Vivo

**Figure 2:**
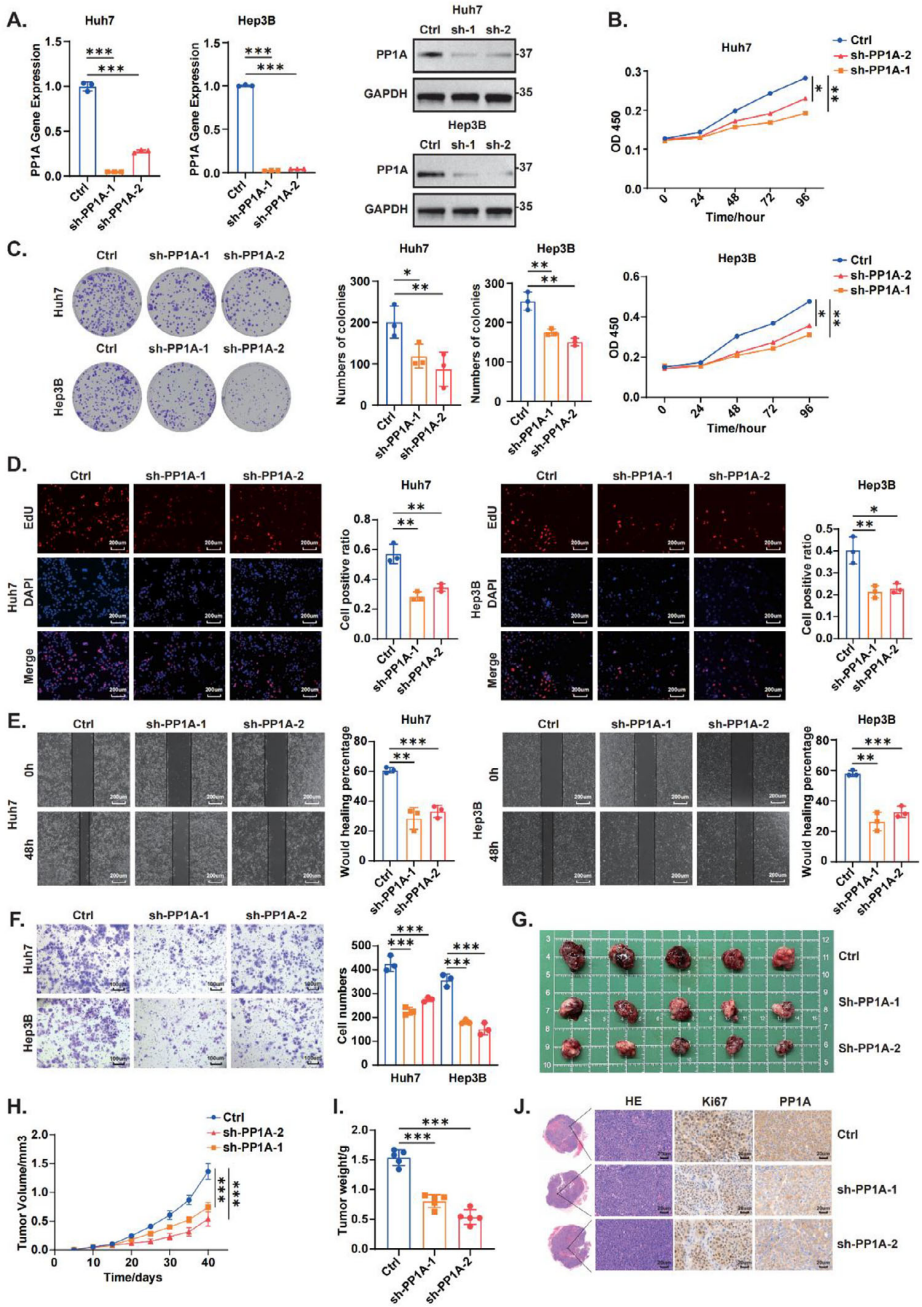
PP1A promotes the proliferation, invasion, and metastasis of HCC cells in vitro and in vivo. A) Validation of PP1A expression following knockdown in the indicated cell lines was conducted using qRT‐PCR and Western blot. B‐C) The proliferation of Huh7 and Hep3B cells was assessed using CCK‐8 and colony formation assays. D) The proliferation of Huh7 and Hep3B cells was also evaluated using EdU assays. E‐F) The migratory and invasive abilities of Huh7 and Hep3B cells were measured using wound healing and transwell assays. G‐I) Representative images of xenograft tumors in nude mice, along with statistical analyses of tumor volumes and tumor weights across the different groups (n = 5). J) Immunohistochemical analysis of Ki67 and PP1A expression in xenograft tumors. One‐way ANOVA was used to analyze the data. ^*^ *p* < 0.05, ^**^ *p* < 0.01, ^***^ *p* < 0.001. n.s, not significant. The data are expressed as the mean±SD of three independent experiments.

We examined PP1A levels in five HCC cell lines and noted high PP1A expression in Huh7 and Hep3B cells, whereas low expression was observed in the normal liver cell line THLE‐2 (Figure S1J, Supporting Information). Next, we designed shRNAs for the construction of PP1A knockdown cell lines (**Figure** **2**A). The results of the CCK‐8 and colony formation assays revealed that PP1A knockdown inhibited cell proliferation (Figure 2B,C), which was further supported by the results of EdU assays (Figure 2D). Wound healing and transwell assays confirmed that PP1A knockdown reduced HCC cell migration and invasion (Figure 2E, F). The overexpression of PP1A (Figure S2A, Supporting Information) promoted cell proliferation (Figure S2B, Supporting Information) and metastasis (Figure S2C, Supporting Information). To further elucidate the role of PP1A in vivo, we established subcutaneous xenograft tumors in nude mice (Figure 2G; Figure S2D, Supporting Information). Consistent with the in vitro results, PP1A knockdown reduced tumor size and weight (Figure 2H,I), whereas PP1A overexpression had the opposite effects (Figure S2E,F, Supporting Information). IHC analysis of subcutaneous xenograft tumor tissues revealed a positive correlation between PP1A expression and Ki67 expression (Figure 2J; Figure S2G, Supporting Information), indicating that it facilitates a role in promoting tumor progression by promoting cell proliferation. We further established an orthotopic HCC model using Hep53.4 cells in C57BL/6 mice, incorporating PP1A‐knockdown and overexpression groups with their respective controls. The results demonstrated the promotive effect of PP1A on tumor growth (Figure S2H,I, Supporting Information). In conclusion, our findings indicate that PP1A promotes tumor growth and metastasis in HCC both in vitro and in vivo.

### 2.3 PP1A Inhibits Lenvatinib‐Induced Ferroptosis in HCC

**Figure 3:**
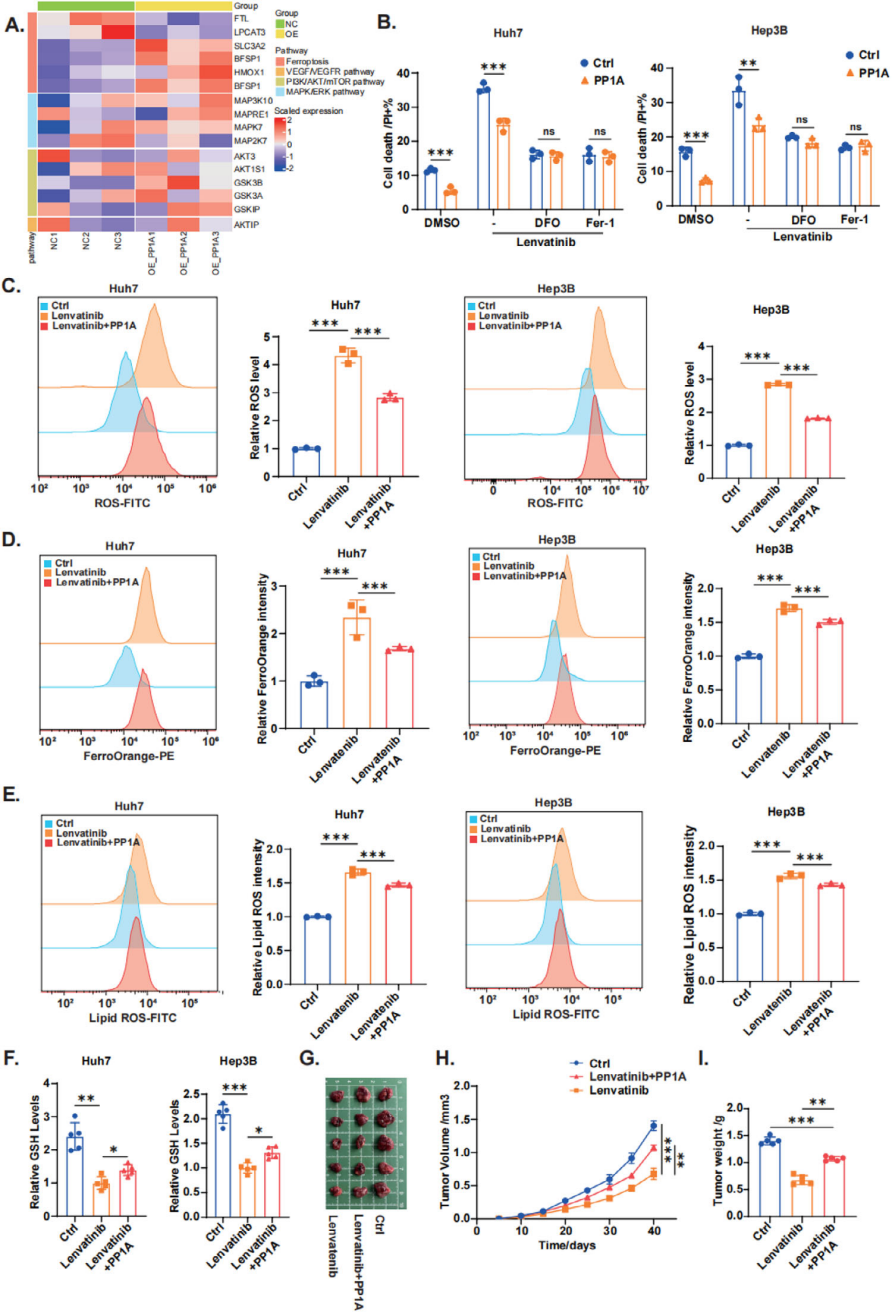
PP1A inhibits Lenvatinib‐induced ferroptosis in HCC. A) Differential gene expression analysis of RNA‐seq data from HCC cells overexpressing PP1A. Upregulated and downregulated genes are highlighted in red and blue, respectively (P < 0.05). B) Apoptosis of Huh7 and Hep3B cells was analyzed by flow cytometry. C) Intracellular ROS levels in Huh7 and Hep3B cells were detected by flow cytometry using DCFH‐DA. D) Intracellular Fe^2+^ levels in Huh7 and Hep3B cells were measured by flow cytometry using FerroOrange. E) Lipid ROS accumulation in Huh7 and Hep3B cells was assessed by flow cytometry using C11‐BODIPY 581/591. F) Intracellular GSH levels in Huh7 and Hep3B cells were quantified using a GSSG/GSH quantification kit. G‐I) Representative images of xenograft tumors in nude mice, along with statistical analyses of tumor volumes and tumor weights across the different groups (n = 5). Unpaired Student's t‐test or one‐way ANOVA was used to analyze the data. ^*^ *p* < 0.05, ^**^ *p* < 0.01, ^***^ *p* < 0.001. n.s, not significant. The data are expressed as the mean±SD of three independent experiments.

To confirm whether PP1A influenced Lenvatinib resistance in HCC cells through ferroptosis, we overexpressed PP1A in HCC cells and conducted RNA sequencing (RNA‐seq). Upon analyzing the pathways involved in Lenvatinib/Sorafenib‐induced cancer cell death, we found that only ferroptosis‐related pathway genes were significantly upregulated (**Figure** **3**A). Additionally, we analyzed the Gepia2 and TIMER2.0 databases and confirmed the correlation between PP1A and key ferroptosis markers in HCC clinical samples (Figure S3A,B, Supporting Information). To verify the specific role of PP1A in ferroptosis, we performed a rescue experiment by reintroducing PP1A into PP1A‐knockdown cells. The results showed that PP1A knockdown increased ROS, Fe^2^⁺, and lipid ROS levels (Figure S4A–C, Supporting Information), and decreased intracellular GSH levels (Figure S4D, Supporting Information). These changes were reversed by the PP1A reintroduction. Next, we assessed the role of PP1A in suppressing Lenvatinib‐induced ferroptosis. Notably, the control cells exhibited markedly increased susceptibility to ferroptosis following Lenvatinib treatment, whereas the PP1A‐overexpressing cells presented notable resistance (Figure 3B). Further analysis of ferroptosis‐associated metabolites confirmed that Lenvatinib increased the levels of ROS, Fe^2+^, and lipid ROS (Figure 3C–E), and decreased the level of intracellular GSH (Figure 3F), whereas PP1A overexpression attenuated these effects. These effects were reproduced in subcutaneous xenograft tumors in nude mice (Figure 3G,H). Moreover, IHC analysis of subcutaneous xenograft tumor tissues revealed that the expression of PP1A was positively correlated with that of GPX4 and NQO1, which are crucial for ferroptosis (Figure S4E, Supporting Information). Collectively, our in vitro and in vivo findings confirmed that PP1A effectively suppresses Lenvatinib‐induced ferroptosis in HCC cells.

### 2.4 PP1A Interacts with Keap1 and Dephosphorylates Keap1 at the S104 site

**Figure 4:**
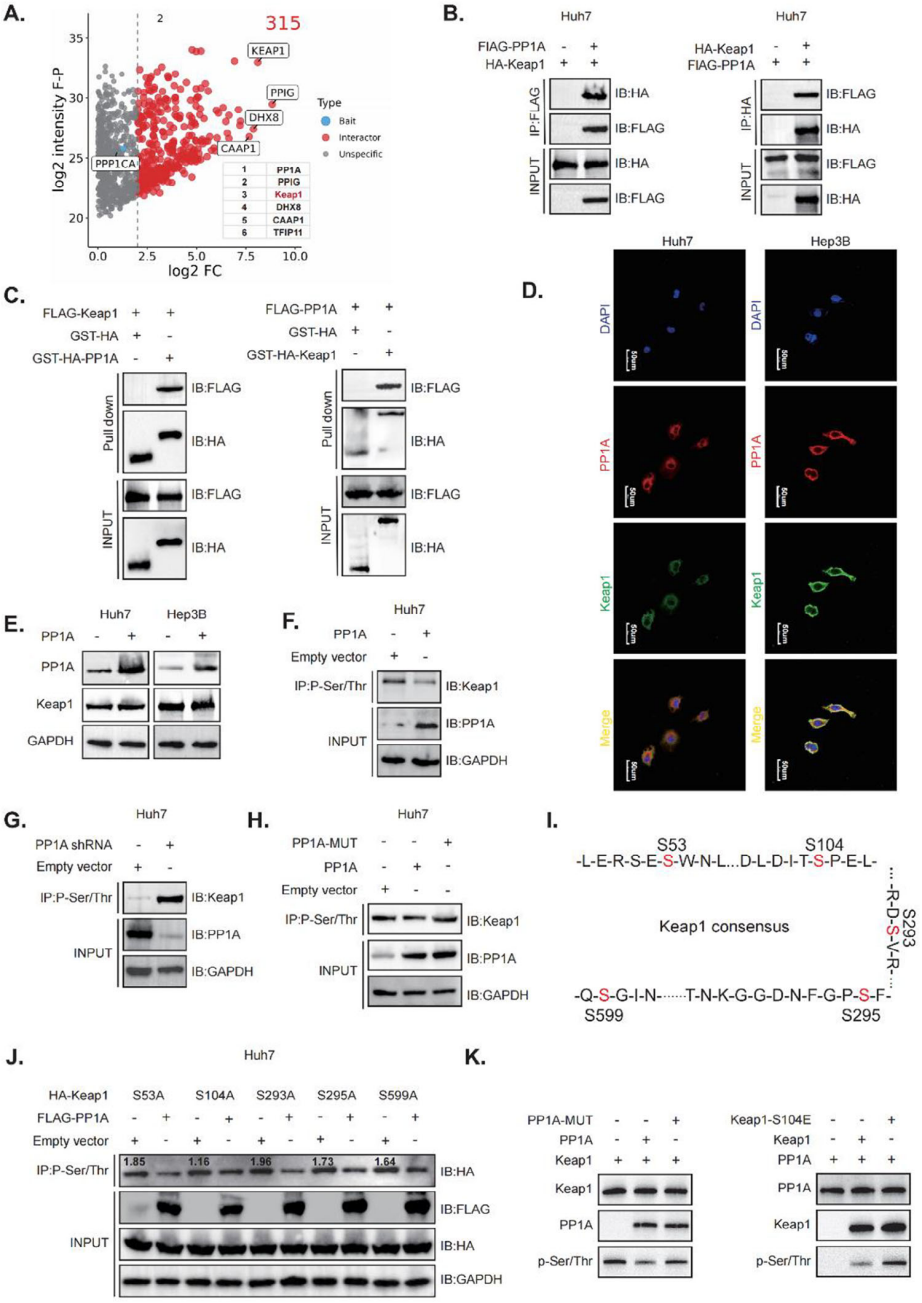
PP1A interacts with Keap1 and dephosphorylates Keap1 at the S104 site. A) PP1A‐interacting proteins were identified through mass spectrometry analysis following immunoprecipitation (IP). B‐C) Co‐immunoprecipitation (Co‐IP) and GST pull‐down assays were conducted to confirm the protein interaction between PP1A and Keap1 in vivo and in vitro, respectively. D) Immunofluorescence assays demonstrated the colocalization of PP1A and Keap1 in Huh7 and Hep3B cells (scale bar, 50um). E) Western blot analysis was used to assess the relationship between PP1A and Keap1 protein levels. F‐G) Phosphorylation IP assays were performed to evaluate the effect of PP1A on Keap1 phosphorylation levels. H) PP1A phosphorylation site mutant plasmids were constructed, and IP assays were conducted to assess the effect of mutated PP1A plasmids on Keap1 phosphorylation levels. I) Five Keap1 phosphorylation sites were predicted using database analysis. J) Keap1 mutant plasmids were constructed to validate phosphorylation via IP assays. K) The Western blot shows the results of PP1A/PP1A‐MUT phosphorylating Keap1/Keap1 mutants in vitro.

To clarify the molecular mechanisms of PP1A in ferroptosis in HCC cells, we carried out immunoprecipitation (IP) of samples from in Huh7 cells and performed mass spectrometry (MS) to identify ferroptosis‐related proteins. We found that Kelch‐like ECH‐associated protein (Keap1) plays an important role in the nuclear factor erythroid 2‐related factor 2 (Nrf2) pathway, regulating oxidative stress and ferroptosis (**Figure** **4**A). Co‐IP and GST pull‐down assays confirmed that PP1A directly interacts with Keap1 (Figure 4B,C). Immunofluorescence assays subsequently verified the colocalization of PP1A and Keap1 in HCC cells (Figure 4D). Molecular docking also provided strong evidence (Figure S5A, Supporting Information). Next, we found that the overexpression of PP1A did not significantly alter Keap1 protein levels (Figure 4E). Thus, we hypothesized that PP1A could display phosphatase activity and dephosphorylates Keap1. We subsequently overexpressed PP1A and performed specific phosphorylated IP experiments. Our results revealed decreased Keap1 phosphorylation following PP1A overexpression (Figure 4F), whereas the opposite effect was observed after PP1A knockdown (Figure 4G). Additionally, we constructed PP1A mutants without phosphatase activity (Figure S5B, Supporting Information). IP assays of hosphorylated proteins demonstrated that mutation of PP1A attenuated the PP1A overexpression‐induced suppression of Keap1 phosphorylation (Figure 4H), supporting that PP1A in modulates Keap1 phosphorylation. We next utilized PhosphoSitePlus and NetPhos3.1 to identify five high‐scoring potential phosphorylation sites (Figure 4I). Mutant plasmids were constructed for IP of phosphorylated protein assays. This analysis revealed that PP1A specifically dephosphorylated Keap1 at the S104 site (Figure 4J). To obtain direct biochemical evidence, we purified PP1A, PP1A‐MUT, Keap1, and Keap1‐S104E proteins in vitro (Figure S5C, Supporting Information) and performed an in vitro phosphorylation assay. The results confirmed that PP1A dephosphorylates Keap1 in vitro. PP1A phosphatase activity mutation and constitutive phosphorylation of Keap1 inhibit this dephosphorylation (Figure 4K). In summary, we found that PP1A dephosphorylates Keap1 at the S104 site via interaction with Keap1.

### 2.5 PP1A‐Mediated Dephosphorylation of Keap1 Stabilizes Nrf2 Protein Levels by Decreasing Interaction of Keap1 and Nrf2

**Figure 5:**
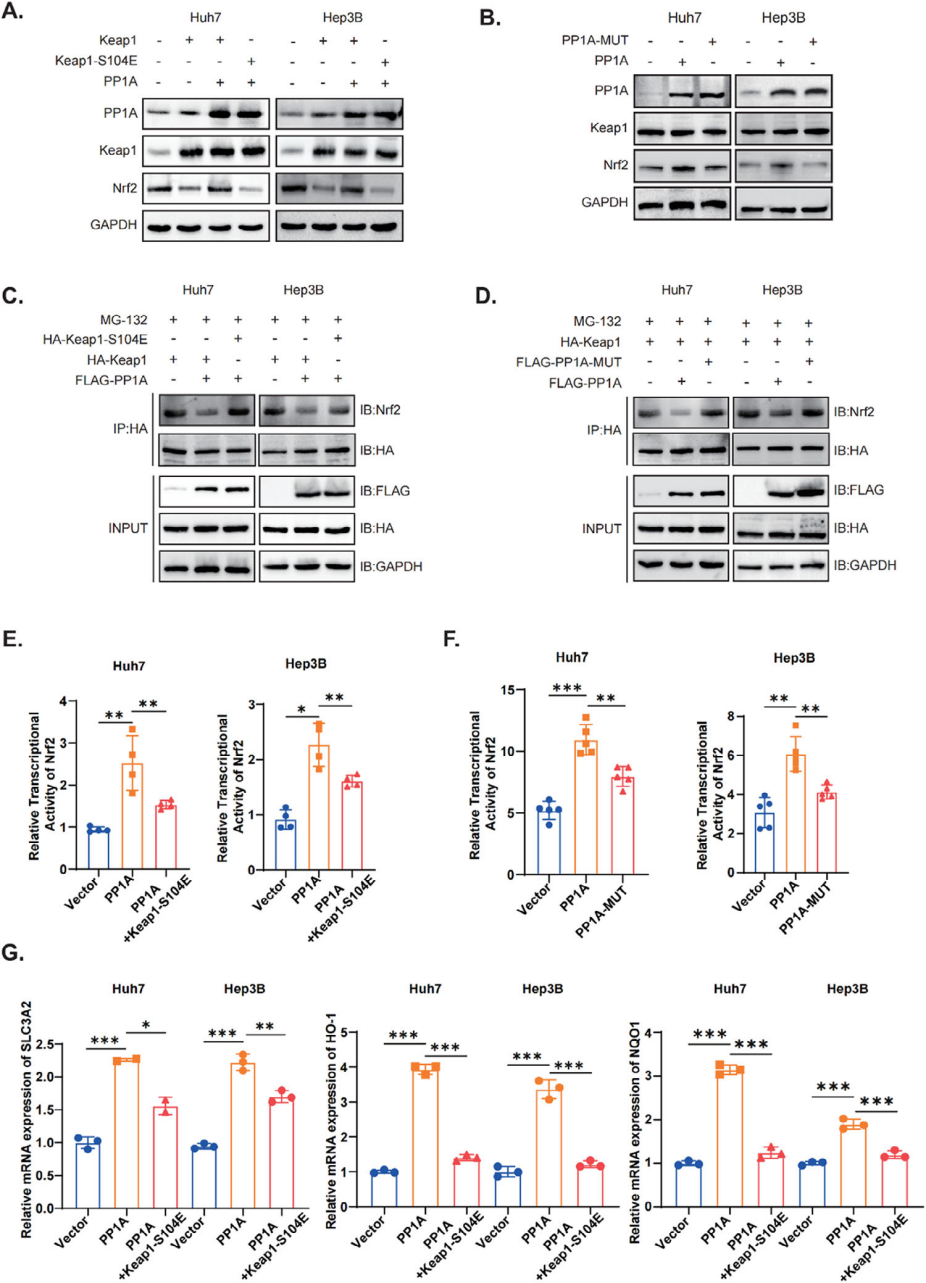
PP1A‐mediated dephosphorylation of Keap1 stabilizes Nrf2 protein levels by decreasing the interaction of Keap1 and Nrf2. A‐B) Western blot analysis was used to evaluate the effects of Keap1 phosphorylation site mutations and PP1A phosphatase activity site mutations on Nrf2 levels, respectively. C‐D) Immunoprecipitation (IP) followed by Western blot was performed to assess the impact of Keap1 phosphorylation site mutations and PP1A phosphatase activity site mutations on the Keap1‐Nrf2 interaction, respectively. E‐F) Dual‐luciferase assays were conducted to examine the effects of Keap1 phosphorylation site mutations and PP1A phosphatase activity site mutations on Nrf2 transcriptional activity, respectively. G) RT‐qPCR was used to detect the influence of Keap1 phosphorylation site mutations on ferroptosis‐related downstream targets of Nrf2. Unpaired Student's t‐test or one‐way ANOVA was used to analyze the data. ^*^ *p* < 0.05, ^**^ *p* < 0.01, ^***^ *p* < 0.001. n.s, not significant. The data are expressed as the mean±SD of three independent experiments.

Previous studies have shown that Keap1 promotes the ubiquitination and proteasomal degradation of Nrf2,^[^ ^17^ ^]^ inhibiting the transcription of downstream ferroptosis‐related genes.^[^ ^18^ ^]^ We therefore hypothesized that PP1A might induce conformational changes in Keap1 through dephosphorylation, resulting in the activation of Nrf2. Thus, we constructed a mutant Keap1 plasmid that mimicked a phosphorylated state. Upregulation of Nrf2 protein expression was observed following PP1A overexpression, and this effect was attenuated by mutations in either the Keap1 phosphorylation site (**Figure** **5**A) or the PP1A phosphatase site (Figure 5B). Additionally, we found that PP1A significantly inhibited the interaction between Nrf2 and Keap1. This effect was also reversed by mutations in either Keap1(Figure 5C) or PP1A (Figure 5D). Similarly, nuclear localization of Nrf2 increased after PP1A overexpression, indicating an upregulating Nrf2 nuclear translocation, and this change was attenuated by mutation in the Keap1 104 site (Figure S5D, Supporting Information). Dual‐luciferase reporter assays were conducted to investigate changes in Nrf2 transcriptional activity. Overexpression of PP1A could enhance the transcriptional activity of Nrf2, which was reduced in the presence of either Keap1 (Figure 5E) or PP1A mutations (Figure 5F). Additionally, we measured the mRNA levels of the Nrf2 downstream ferroptosis‐related genes SLC3A2, HO‐1, and NQO1. The results indicated that these genes were upregulated in response to PP1A overexpression, and these effects were markedly attenuated by mutations in Keap1 (Figure 5G). In conclusion, our findings validated that PP1A stabilized Nrf2 by dephosphorylating Keap1, thereby activating the Nrf2 antioxidant pathway.

### 2.6 Mutations in Keap1 and PP1A Attenuate Ferroptosis Reduced by PP1A Overexpression

**Figure 6:**
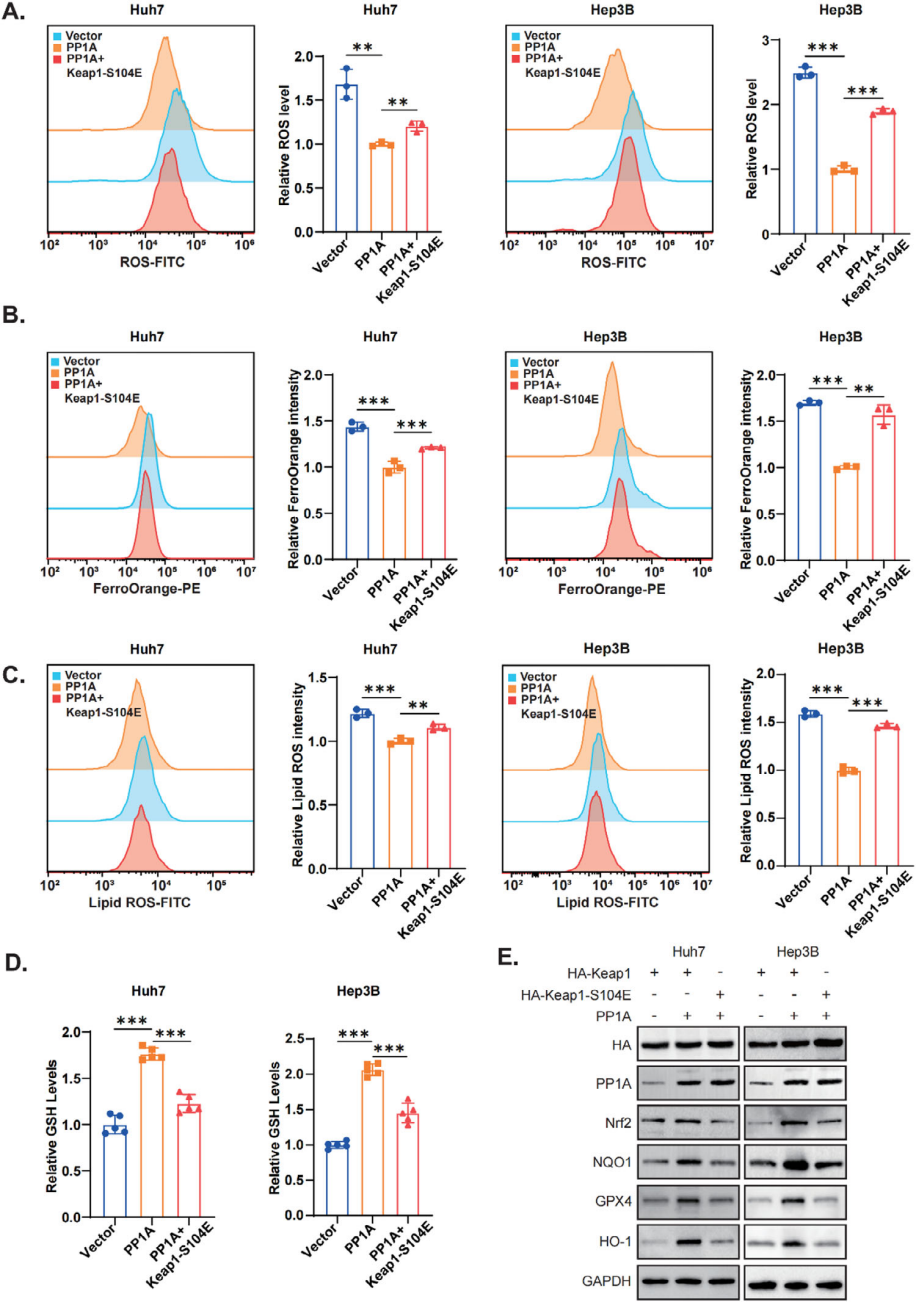
Mutations in Keap1 and PP1A attenuate ferroptosis induced by PP1A overexpression. A) Intracellular ROS levels in Huh7 and Hep3B cells were detected by flow cytometry using DCFH‐DA. B) Intracellular Fe^2^⁺ levels in Huh7 and Hep3B cells were measured by flow cytometry using FerroOrange. C) Lipid ROS accumulation in Huh7 and Hep3B cells was assessed by flow cytometry using C11‐BODIPY 581/591. D) Intracellular GSH levels in Huh7 and Hep3B cells were quantified using a GSSG/GSH quantification kit. E) Western blot analysis was performed to detect the expression of ferroptosis‐related downstream proteins. Unpaired Student's t‐test or one‐way ANOVA was used to analyze the data. ^*^ *p* < 0.05, ^**^ *p* < 0.01, ^***^ *p* < 0.001. n.s, not significant. The data are expressed as the mean±SD of three independent experiments.

To further confirm that PP1A inhibits ferroptosis through Keap1 dephosphorylation, we conducted rescue experiments by mutating Keap1. We found that mutation of Keap1 reversed the protective effect of PP1A overexpression against ferroptosis in HCC cells. Comparable results were observed in intracellular ROS levels (**Figure** **6**A), Fe^2^⁺ concentrations (Figure 6B), lipid ROS accumulation (Figure 6C), and GSH levels (Figure 6D). Additionally, Western blot analysis of downstream ferroptosis‐related genes revealed consistent results (Figure 6E). Similarly, experiments were conducted to assess the effects of mutations in PP1A. Loss of PP1A phosphatase activity reversed the protective effect of PP1A overexpression against ferroptosis. We observed the same results in intracellular ROS levels (Figure S6A, Supporting Information), Fe^2^⁺ concentrations (Figure S6B, Supporting Information), lipid ROS accumulation (Figure S6C, Supporting Information), and GSH levels (Figure S6D, Supporting Information). Western blot analysis of downstream ferroptosis‐related genes revealed consistent outcomes (Figure S6E, Supporting Information). Our results suggested that both Keap1 and PP1A mutations effectively attenuate the protection against ferroptosis conferred by PP1A overexpression.

### 2.7 PP1A Enhances Tumor Immunity by Upregulating PD‐L1 and Increases the Efficacy of Lenvatinib Plus ICIs

Recent studies underscore the role of ferroptosis in antitumor immunity, particularly in T cell mediated immune responses.^[^ ^19^ ^]^ Additionally, Nrf2 regulates the expression of PD‐L1, a component of the PD‐1/PD‐L1 axis essential for tumor immune evasion,^[^ ^20^ ^]^ suggesting that Nrf2 facilitates immune evasion by upregulating PD‐L1. Bioinformatics analysis revealed a correlation between PP1A expression and immune infiltration (Figure S7A, Supporting Information).

**Figure 7:**
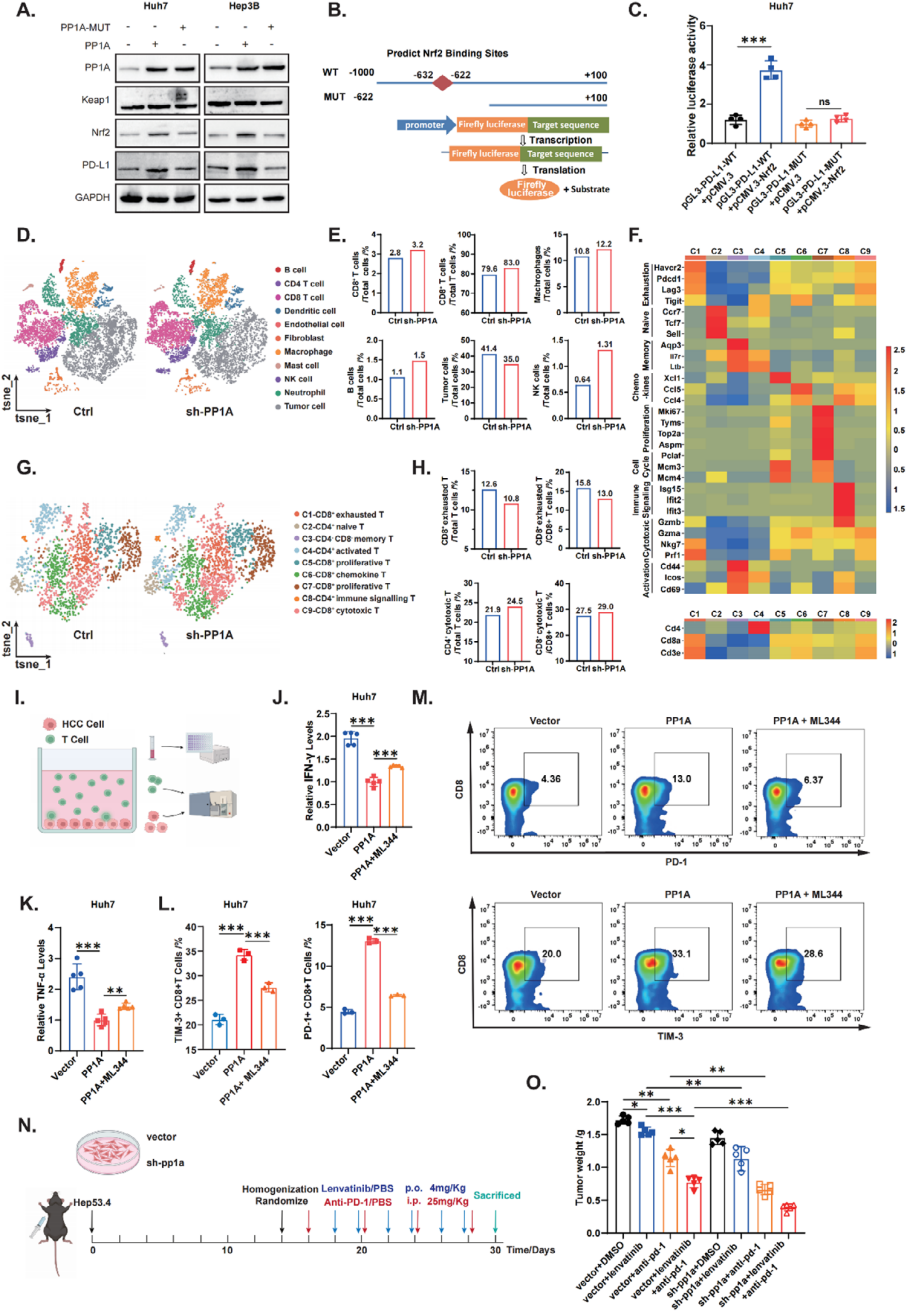
PP1A enhances tumor immunity by upregulating PD‐L1 and increases the efficacy of Lenvatinib plus ICIs. A) Western blot analysis of the effects of PP1A and PP1A mutant plasmids on PD‐L1 protein expression. B‐C) Dual‐luciferase reporter assays to assess Nrf2 regulation of PD‐L1 transcription levels. D‐E) Single‐cell sequencing was performed on tumor samples from Ctrl and sh‐PP1A mice models, followed by clustering and comparison of the cells. F) Heatmap analysis revealed the specifically high‐expressed genes in each T cell subcluster G‐H) TSNE maps and proportion of T cell subclusters I) Schematic Diagram of the Co‐culture Experiment of HCC Cells and T Cells. J‐K) Co‐culture of HCC cells and T cells, followed by ELISA to measure TNF‐α and IFN‐γ cytokine levels in the supernatant. L‐M) Flow cytometry analysis of the proportion of Exhausted CD8+ T cells in suspended T cells. N) Experimental design and group assignments for the animal study. O) Measurement and analysis of syngeneic tumor weights (n = 5). Unpaired Student's t‐test or one‐way ANOVA was used to analyze the data. Details of the synergistic effect calculations are provided in Supplementary Table S4 (Supporting Information). ^*^ *p* < 0.05, ^**^ *p* < 0.01, ^***^ *p* < 0.001. n.s, not significant. The data are expressed as the mean ± SD of three independent experiments. i.p., intraperitoneal injection. p.o. per os.

An initial analysis was conducted via Gepia2 and cBioPortal to assess the correlation between PP1A and PD‐L1 expression in clinical HCC samples (Figure S7B, Supporting Information). We next performed a rescue experiment by reintroducing PP1A into PP1A‐knockdown cells to verify the specific role of PP1A in PD‐L1 regulation. The results showed that PP1A knockdown led to decreased levels of PD‐L1, which was reversed by PP1A reintroduction (Figure S8A, Supporting Information). Consequently, we hypothesized that PP1A might influence antitumor immunity by regulating PD‐L1 expression through the Keap1‐Nrf2 pathway. Western blot analysis revealed increased PD‐L1 levels after the overexpression of PP1A, which was reversed by PP1A mutation (**Figure** **7**A). qRT‐PCR further confirmed the relation between PP1A and PD‐L1 at the mRNA level (Figure S8B, Supporting Information). We utilized the JASPAR database to predict potential Nrf2 binding sites and selected the candidates with the highest scores (Figure 7B). Dual‐luciferase reporter assays (Figure 7C; Figure S8C, Supporting Information) and chromatin immunoprecipitation (Figure S8D,E, Supporting Information) confirmed the correlation between Nrf2 and PD‐L1. Moreover, we detected an increase in cell surface PD‐L1 expression after PP1A overexpression, which was reversed by the Nrf2 pathway inhibitor ML344 (Figure S8F, Supporting Information). To further evaluate the role of PP1A in immunotherapy, particularly in the TME of HCC, we established subcutaneous syngeneic tumor models using Hep53.4 cells in C57/BL6 mice and carried out single‐cell sequencing. The t‐SNE plot revealed eleven common cell clusters, which were named according to marker identification and mergered (Figure 7D). We found that the proportion of CD8^+^ T cells increased after PP1A knockdown, increases in the proportions of B cells, macrophages, and NK cells, and a decrease in the proportion of tumor cells were also observed (Figure 7E). Next, we performed unsupervised clustering of all the T cells. Histograms and heatmaps revealed the specific expression of each subcluster (Figure 7F,G). The proportion of exhausted CD8^+^T cells decreased following PP1A knockdown, whereas CD4^+^ cytotoxic T cells increased (Figure 7H). Next, we performed unsupervised clustering of all T cells. The classification of T cell subclusters, particularly CD8^+^ T cells, is annotated on the right side of Figure 7G. Each cluster corresponds to a specific T cell subset. The classification criteria are presented in the heatmap in Figure 7F. The left axis lists nine T cell subpopulations and their markers (Figure S9A,B, Supporting Information). This classification is based on a study published in 2024.^[^ ^21^ ^]^ IHC results showed that PP1A knockdown in the orthotopic HCC model led to lower PD‐L1 expression and higher CD8⁺ T cell infiltration, while PP1A overexpression had the opposite effect (Figure S10A,B, Supporting Information).

To further explore the effects of PP1A on T cells, Huh7 cells were cocultured with T cells from healthy donors (Figure 7I), and the supernatants were subsequently analyzed by enzyme‐linked immunosorbent assay (ELISA). Decreased levels of the cytokines TNF‐α and IFN‐γ were observed when PP1A was overexpressed, and these decreases were reversed by inhibition of the Nrf2 pathway (Figure 7J,K). Additionally, we found that the proportion of exhausted CD8^+^ T cells increased after the overexpression of PP1A, and this increase was reversed by the inhibition of the Nrf2 pathway (Figure 7L,M). These findings confirmed the role of PP1A in promoting tumor immune resistance through the Nrf2 pathway. Given the demonstrated protective effect of PP1A on Lenvatinib‐induced ferroptosis, we aimed to investigate whether PP1A can enhance the efficacy of Lenvatinib plus ICIs therapy. Anti‐PD‐1 monotherapy and Lenvatinib were used in the next experiment. We established subcutaneous syngeneic tumor models in C57BL/6 mice using control or PP1A knockdown Hep53.4 cells, which were randomized to receive different treatments (Figure 7N). We first confirmed the efficacy of combination therapy compared with monotherapy. More importantly, the results demonstrated that PP1A knockdown significantly increased the efficacy of combination therapy (Figure 7O; Figure S8G, Supporting Information). Immunofluorescence staining showed that PP1A knockdown significantly enhanced CD8^⁺^ cell infiltration in both the ICI monotherapy and combination therapy groups (Figure S11A, Supporting Information). TUNEL staining further confirmed that PP1A knockdown enhanced drug‐induced apoptosis, particularly in the combination treatment group of ICI and Lenvatinib (Figure S11A, Supporting Information). Therefore, we demonstrated that targeting PP1A can enhance antitumor immunity. Moreover, our findings suggest that PP1A can serve as a potential treatment target and efficacy predictor for Lenvatinib plus ICIs therapy.

## 3 Discussion

Lenvatinib is crucial in treating HCC and serves as an alternative to Sorafenib.^[^ ^4^, ^5^ ^]^ The REFLECT trial in HCC showed that Lenvatinib was non‐inferior to Sorafenib regarding overall survival (OS) and improved all secondary efficacy endpoints.^[^ ^22^ ^]^ However, over 60% of HCC patients develop resistance to Lenvatinib within a year, with only a few attaining long‐term benefits.^[^ ^23^ ^]^ Therefore, understanding the mechanisms of Lenvatinib resistance and identifying effective solutions are critical. Previous research has identified elevated PP1A expression in Lenvatinib‐ and Sorafenib‐resistant cells (Figure S1E,F, Supporting Information). PP1A also promotes tumor progression in various malignancies.^[^ ^15^, ^16^ ^]^ We next confirmed that PP1A was upregulated in HCC and associated with poor prognosis (Figure 1C). Loss‐ and gain‐of‐function experiments both in vitro and in vivo validated its oncogenic potential (Figure 2B–J; Figure S2B–G, Supporting Information).

Recent studies found that Lenvatinib suppresses GPX4 expression, leading to the accumulation of lipid ROS and ferroptosis.^[^ ^12b^ ^]^ Ferroptosis is a ROS‐dependent form of cell death that may provide new opportunities for cancer treatment. Post‐translational modifications, such as phosphorylation, have been proven to be closely related to ferroptosis.^[^ ^24^ ^]^ However, the mechanisms by which phosphorylation regulates ferroptosis in tumor progression and treatment resistance remain unclear. Bioinformatics analysis revealed a correlation between PP1A expression and ferroptosis‐related markers (Figure S3, Supporting Information). To further elucidate the role of PP1A in ferroptosis, we conducted RNA‐seq and metabolite assays, revealing that PP1A inhibited Lenvatinib‐induced ferroptosis in HCC cells (Figure 3). Further investigation indicated that PP1A modulated the Keap1‐Nrf2 pathway by dephosphorylating Keap1 (Figure 4B–H). The Keap1‐Nrf2 pathway is crucial to cell defense and antioxidant response. High expression of Nrf2 in cancer cell lines shows more resistance to a variety of anti‐cancer drugs. An important mechanism for activating Nrf2 is disrupting the interaction between Nrf2 and Keap1. Previous studies have shown that Keap1 regulates Nrf2 by promoting its ubiquitination and proteasomal degradation.^[^ ^17^ ^]^ During oxidative stress, amino acids in Keap1 undergo covalent modifications, therefore leading to a structural change and allowing Nrf2 to evade Keap1‐mediated ubiquitination, translocate to the nucleus^[^ ^25^ ^]^ and start the transcription of genes that protect against oxidative damage and ferroptosis.^[^ ^18^ ^]^ Rescue experiments confirmed that PP1A exerted its phosphatase function to regulate the Nrf2 pathway, affecting Nrf2 transcriptional activity and downstream ferroptosis‐related markers, such as expression of GPX4, NQO1, and HO‐1, thereby protecting HCC cells from ferroptosis (Figure 6). Additionally, PP1A was found to influence Keap1 phosphorylation at the S104 site (Figure 4I–K), altering its conformation and interaction with Nrf2 (Figure 5). These results are consistent with the previously reported regulatory relationship between Keap1 and Nrf2.^[^ ^17^ ^]^ Collectively, our study suggested that PP1A inhibited ferroptosis through its regulation of the Nrf2 pathway, facilitated by its phosphatase activity in HCC.

Previous studies have shown that Nrf2 promotes PD‐L1 transcription by binding to its promoter region.^[^ ^20^ ^]^ The PD‐1/PD‐L1 pathway, where PD‐1 interacts with PD‐L1 on tumor cells, enables immune escape, providing significant breakthroughs for HCC treatment.^[^ ^26^ ^]^ ICIs, such as anti‐PD‐1, have shown strong antitumor activity in some HCC patients.^[^ ^27^ ^]^ In recent years, immunotherapy has become a leading treatment for HCC.^[^ ^28^ ^]^ However, at least 30% of HCC tumors exhibit intrinsic resistance to PD‐1 or PD‐L1 inhibitors.^[^ ^9^ ^]^ Phase III trials have proved that the antitumor efficacy of ICIs as single agents was insufficient to enhance OS in treatment‐naïve patients.^[^ ^29^ ^]^ Increasing evidence suggested that the clinical response to anti‐PD‐1/PD‐L1 therapy is strongly correlated with PD‐L1 expression on tumor cells and T lymphocyte infiltration.^[^ ^10^ ^]^ Inducing ferroptosis has been found to enhance antitumor immune responses by promoting CD8^+^ T cell infiltration and cytotoxicity, as well as activating immune cells within the TME,^[^ ^30^ ^]^ significantly enhancing the efficacy of immunotherapy. CD8^+^ T cells activated by immunotherapy can trigger ferroptosis in tumor cells through IFN‐γ secretion, enhancing the cytotoxic effect on cancer cells.^[^ ^31^ ^]^ A comprehensive understanding of the mechanisms that regulate PD‐L1 expression and T lymphocyte infiltration may help develop novel therapeutic strategies to improve the efficacy of PD‐1/PD‐L1 blockade.

A recent study found that reducing PD‐L1 expression can enhance the effectiveness of PD‐1 monoclonal antibody treatment by improving the blocking of the PD‐1/PD‐L1 interaction, thereby reducing immune evasion, alleviating immune suppression, and modulating the tumor microenvironment.^[^ ^28^, ^32^ ^]^ Bioinformatics analysis revealed a correlation between PP1A expression, immune infiltration, and PD‐L1 expression. (Figure S7A,B, Supporting Information). Our experiments further confirmed that PP1A regulated PD‐L1 expression through the Nrf2 pathway (Figure 7A–C). Single‐cell sequencing demonstrated that PP1A knockdown reduced exhausted CD8^+^ T cells, indicating its role in T cell exhaustion. PP1A knockdown also increased macrophage and B cell infiltration while decreasing tumor cell infiltration, suggesting its impact on the TME (Figure 7D–H). In vitro, co‐culture experiments demonstrated that upregulation of PP1A not only inhibited ferroptosis but also enhanced CD8^+^ T cell infiltration and cytotoxicity (Figure 7I–M). Since PP1A could affect ferroptosis and induce immune evasion through PD‐L1, we hypothesized that PP1A might bridge the ferroptosis and antitumor immunity in HCC TME, potentially enhancing the efficacy of combination therapy.

**Figure 8:**
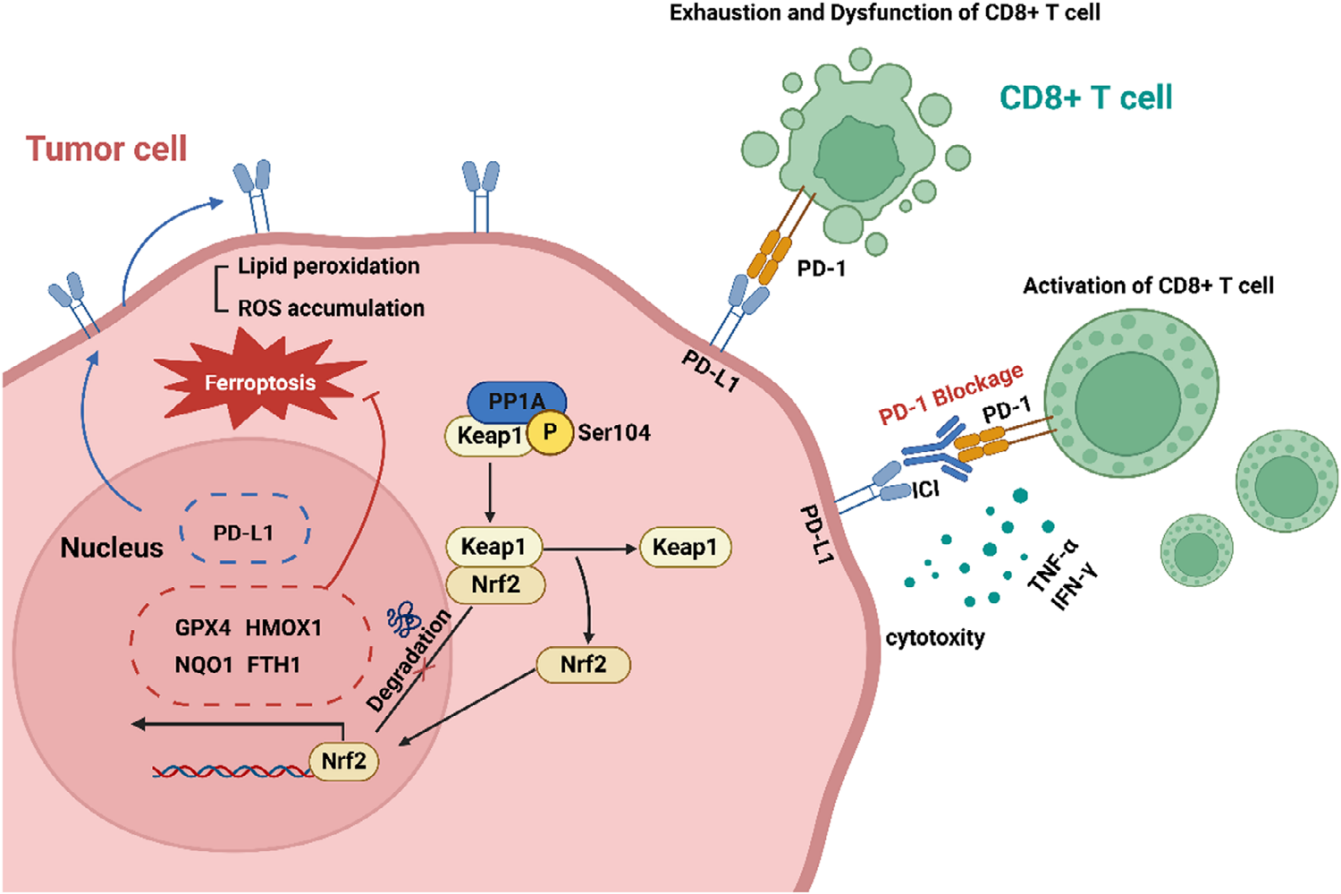
A model illustrating the role of PP1A in regulating ferroptosis and antitumor immunity in HCC cells. PP1A dephosphorylate2s Keap1 at the S104 site, stabilizing and promoting the nuclear translocation of Nrf2, which upregulates the expression of antioxidant genes and PD‐L1. This leads to suppressed ferroptosis and enhanced immune evasion, thereby improving the efficacy of TKIs and ICIs combination therapy.

In recent years, combination therapy has emerged as a promising strategy for patients unresponsive to either Lenvatinib or ICIs monotherapy. Clinical studies indicate that the combination of Lenvatinib and ICIs therapy improves OS and progression‐free survival (PFS) in advanced HCC patients, demonstrating superior efficacy and fewer side effects compared to monotherapy.^[^ ^33^ ^]^ The combination of Lenvatinib and ICIs therefore represents a promising avenue.^[^ ^26^ ^]^ We further demonstrated that targeting PP1A enhanced the therapeutic efficacy of Lenvatinib plus ICIs therapy in vivo. The combination shows significantly better efficacy compared to either Lenvatinib or ICIs monotherapy, consistent with clinical results.^[^ ^33b^ ^]^ We also observed enhanced monotherapy efficacy after PP1A knockdown. More importantly, our results showed that PP1A knockdown notably enhanced the efficacy of combination therapy (Figure 7N,O). These findings provide novel molecular targets and theoretical insights for improving the efficacy of Lenvatinib plus ICIs therapy, emphasizing the potential of developing strategies that combine ferroptosis inducers with ICIs in HCC and offering a new perspective for combination therapy in HCC (**Figure** **8**).

In conclusion, our study demonstrated that upregulation of PP1A inhibited ferroptosis and CD8^+^ T cell‐mediated antitumor immunity in HCC. Notably, PP1A played a critical role in the therapeutic efficacy of the combination of Lenvatinib and ICIs. This finding suggested that PP1A could serve as a valuable target for evaluating and potentially enhancing the effectiveness of combination treatment strategies in HCC.

## 4 Experimental Section

### Tissue Samples and Cell Culture

HCC samples and paired nontumor liver tissue samples were obtained from the patients hospitalized at the Zhongnan Hospital, Wuhan University (Hubei Province, China). Written informed consent was obtained from all patients prior to the collection and use of their tissues in this study. The study was conducted with approval from the Research Ethics Committee of the Second Affiliated Hospital with Wuhan University School of Medicine (2022181K). All samples were collected with the informed consent of the patients according to the guidelines of the 1975 Declaration of Helsinki. The harvested tissues were immediately frozen and stored for subsequent qRT‐PCR and Western blot analyses. All cell lines used in the study were procured from the Chinese Type Culture Collection. These cell lines were cultured in Dulbecco's modified Eagle's medium (DMEM)/high glucose (GE, USA), supplemented with 10% fetal bovine serum (FBS, Gibco, USA), and maintained at 37°C in a humidified incubator under 5% CO2 conditions.

### Single‐Cell Sequencing

Single‐cell capture was performed using a BD Rhapsody system. Whole transcriptome libraries were generated following the BD Rhapsody single‐cell whole transcriptome amplification workflow. Sequencing was carried out on a HiSeq Xten (Illumina, San Diego, California, USA) platform, with employing 150 bp paired‐end reads.

### ScRNA‐Seq Data Processing

The scRNA‐seq data of all the samples were normalized by using the Seurat package (version 3.0) in R (version 3.5.3). Genes expressed in fewer than three cells in a sample were excluded, as were cells that expressed fewer than 500 genes or whose mitochondrial gene content was greater than 20% of the total UMI count. The total number of transcripts in every single cell was normalized to 10,000. Highly variable genes, namely genes with very different levels of expression in different cells, were detected according to the average expression (between 0.05 to 3) and dispersion (above 0.5) of the genes; this step was followed by data scaling (subtracting the average expression) and centering (dividing standard deviation). These variable genes were considered to account for cell‐to‐cell differences, and were further used for principal component analysis (PCA). The first 25 principal components (PCs) were applied for t‐SNE analysis according to the eigenvalues.

### Cell Coculture Assay

Peripheral venous blood (6 ml) was collected from healthy volunteers using heparin anticoagulant tubes. The blood was then diluted with Ficoll lymphocyte separation solution (Sigma, American). Peripheral blood mononuclear cells (PBMCs) were isolated via gradient density centrifugation and subsequently counted. The EasySep™ T cell isolation kits (STEMCELL Technologies, Canada) were used for the separation of T cells. Huh7 cells were cultured in fresh medium and cocultured with activated T cells at a ratio of 1:5 (cancer cells: CIK cells) for 48 h. After incubation, the plates were centrifuged at 400 g for 5 min, and the supernatant was collected. A lactate dehydrogenase (LDH) release assay (Beyotime China) was performed according to the manufacturer's instructions, and absorbance was measured at 490 nm using a Biotek microplate reader. Finally, IFN‐γ and TNF‐α concentrations in the supernatant were analyzed by enzyme‐linked immunosorbent assay (ELISA) (R&D Systems, Minnesota, USA) following the manufacturer's guidelines.

### Plasmids and shRNA Transfection

The full‐length or mutant PP1A and Keap1 genes were amplified via PCR and inserted into the pcDNA5 vector. The plasmids were transfected into HEK‐293T, Huh7, and Hep3B cells using Lipo3000 (Thermo Fisher, NY, USA). Some of the related plasmids were designed by Qingke Biological (Wuhan, China), while others were purchased from Miaoling (Wuhan, China). The negative control and shRNA constructs were packaged into lentiviruses. All sequences of primers used to construct the plasmids and shRNAs are listed in Supplementary Table S3 (Supporting Information). The transduced cells were then treated with 2 µg/ml puromycin to establish stable PP1A knockdown cell lines.

### Reporter Vector Construction and Luciferase Reporter Assay

A dual‐luciferase reporter assay was used to investigate the interaction between PP1A and the PD‐L1 promoter region. PP1A wild‐type (WT) and mutant (MUT, containing mutated binding sites) sequences were synthesized and inserted into the pGL3‐basic vector. Transfection was performed via Lipofectamine 3000. To assess the activity of these constructs, cell lysates were prepared 48 h post‐transfection, and the activities of firefly and Renilla luciferases were sequentially measured using a Dual‐Luciferase Reporter Assay System (Promega). Firefly luciferase activity was first measured by adding a specific substrate (Luciferase Assay Reagent), followed by termination of the reaction. Renilla luciferase activity was then detected by adding a second substrate (Stop & Glo® Reagent).

### Animal Models

Four‐week‐old BALB/c nude mice and C57BL/6 mice were purchased from Wuhan University Experimental Animal Centre (Wuhan, China). All mice were bred and maintained under specific pathogen‐free (SPF) conditions. Control or two types of stable PP1A knockdown Huh7 cells were subcutaneously injected into the left axillary or tail vein of BALB/c nude mice. The mice were then randomly divided into groups (five mice per group) using a random number table. The sample size was estimated to allow the detection of significant differences between groups. Throughout the experiment, the investigator remained blinded to the group allocation, and tumor size was measured every five days. Forty days after cell injection, the mice were sacrificed, and the tumors were collected and weighed. For orthotopic tumor models, 4 × 10^5^ HCC cells Hep53.4 cells suspended in 0.03 mL Matrigel were injected under the left liver capsule of anesthetized C57BL/6 mice. After injection 2 weeks, mice were sacrificed and livers were collected for histological analyses.

For the C57BL/6 mouse tumor syngeneic model, 1 × 10^6^ Hep53.4 cells suspended in 0.25 mL of Matrigel were injected into the axillary region of anaesthetized C57BL/6 mice. Tumor volumes were monitored every 2 days. When tumor volume reached to 100mm3, 20 mice with tumors of ≈100 mm^3^were selected from the Ctrl and Sh‐PP1A groups and randomly divided into 4 groups. The mice were treated with various agents, including Lenvatinib (MCE, China), anti‐PD‐1 (BioXcell, USA), PBS, and mouse IgG (control; BioXcell, USA). The specific groupings and treatment protocols are shown in Figure 7N. Mice requiring PD‐1 treatment were given anti‐PD1 antibody (BioXcell, USA) (25 mg kg^−1^) intraperitoneally (i.p.) every 4 days. Mice requiring Lenvatinib treatment were treated with Lenvatinib (4 mg kg^−1^) via oral gavage (p.o.) every 2 days. Tumor size was monitored every four days. The animal study was approved by the Ethics Committee of Zhongnan Hospital (WP2023‐0308).

### Chromatin Immunoprecipitation

To investigate the potential binding between the PD‐L1 promoter region and Nrf2 in HCC cells, a chromatin immunoprecipitation assay was performed following exogenous transfection of the Flag‐Nrf2 plasmid. The cells were fixed with formaldehyde, and the lysates were sonicated. The sonicated samples were divided into two groups [an IgG group incubated with 10 µL of IgG antibody (Proteintech, 30000‐0‐AP, China) and an IP group incubated with 10 µL of Flag antibody (Proteintech, 66008‐4‐Ig, China)] for immunoprecipitation. After elution and reverse crosslinking, qRT‐PCR analysis was conducted. All primer sequences are listed in Supplementary Table S1 (Supporting Information).

### Generation of Sorafenib‐Resistant and Lenvatinib‐Resistant Cells

Determine the IC50 values of Sorafenib and Lenvatinib in Huh7 and Hep3B cell lines. The IC50 values for Huh7 are 12 µmol L^−1^ for Sorafenib and 20 µmol L^−1^ for Lenvatinib, while those for Hep3B are 7 and 16 µmol L^−1^, respectively. Culture Huh7 and Hep3B cells at 50% of the IC50 concentration, replacing the culture medium every 72 h. Upon reaching 90% confluence, passage the cells and increase the drug concentration by 1 µmol L^−1^ after replating. Reassess IC50 at every 10 µmol L^−1^ increment. After the third assessment, verify cell line identity and determine the final IC50 (Figure S12A,B, Supporting Information). Sorafenib and Lenvatinib were obtained from MCE (Shanghai, China).

### Co‐Immunoprecipitation (co‐IP) and LC‐MS/MS Analysis

The cells were lysed in IP/Western lysis buffer (Beyotime, China) containing a proteinase and phosphatase inhibitor cocktail (Selleck, USA). The cell lysates were incubated with antibodies in a rotating incubator overnight at 4°C, followed by coincubation with protein A/G magnetic beads (Bimake, China) at 4°C for 6 h. The beads were washed with PBST and boiled in 1×SDS loading buffer (GenScript, China), and the binding proteins were analyzed via LC‒MS/MS or Western blot analysis. Protein samples were then analyzed using a Q Exactive Plus mass spectrometer (Thermo Scientific, Massachusetts, USA) in conjunction with the UltiMate 3000 RSLC nanosystem (Thermo Fisher) for detailed protein identification and quantification.

### Glutathione S transferase (GST) pull‐down assay

A GST pull‐down assay was performed in HEK293 cells because of the high transfection efficiency in these cells. The FLAG‐PP1A plasmid was transfected into HEK293 cells using Lipo 3000. GST‐HA‐Keap1 or control GST‐HA was also transfected simultaneously. A GST‐pulldown assay was subsequently performed using glutathione beads (Tiandirenhe, China), followed by Western blot analysis of the results. Similar GST‐pulldown assays involving for cells transfected with FLAG‐Keap1 and GST‐HA‐PP1A were conducted in the same manner.

### Assessment of intracellular ROS, iron, and lipid peroxidation levels

Cells (1×10⁵ cells well^−1^) were seeded in 12‐well plates and treated for the indicated durations. To detect reactive oxygen species (ROS), iron, and lipid peroxidation levels, cells were incubated in a serum‐free medium with DCFH‐DA (Beyotime, China), FerroOrange (Dojindo, Japan), and C11‐BODIPY 581/591 (Dojindo, Japan). DCFH‐DA (10 µmol L^−1^) was used to detect ROS, with cells incubated in the dark for 30 min. FerroOrange (1 µmol L^−1^) was used to detect Fe^2^⁺, following pretreatment with 10 mmol L^−1^ ammonium ferrous sulfate for 30 min. C11‐BODIPY 581/591 was used to detect lipid ROS, with cells first incubated with 1 mol L^−1^ Liperfluo for 30 min, followed by treatment with 500 mol L^−1^ t‐BHP for 60 min. After incubation, cells were washed three times with PBS or serum‐free medium, digested into a single‐cell suspension, and analyzed by flow cytometry (BD FACSCanto II, USA).

### GSH/GSSG assay

After washing with PBS, equal numbers of cells were collected using a Vi‐Cell cell counter (Beckman Coulter, USA). The intracellular total glutathione and oxidized glutathione disulfide (GSSG) levels were measured using a GSH/GSSG assay kit (Beyotime, China) following the manufacturer's instructions. Absorbance was measured at 405 nm. Standard curves for GSSG and total glutathione (GSH + GSSG) were generated to determine their concentrations. The GSH level was calculated as: GSH = Total Glutathione – 2 × GSSG.

### Statistical Analysis

All data were presented as mean ± standard deviation (SD) unless otherwise noted. Statistical comparisons between the two groups were conducted using a two‐tailed unpaired Student's *t*‐test. For comparisons among three or more groups, a one‐way analysis of variance (ANOVA) followed by appropriate post hoc tests was applied. Dose–response curves and IC₅₀ values were calculated using nonlinear regression analysis in GraphPad Prism (version 9.0.2; GraphPad Software Inc., San Diego, CA, USA). Correlations between variables were evaluated using Pearson's correlation coefficient. Survival analyses were performed using the Kaplan–Meier method and differences between groups were assessed using the log‐rank test. All statistical analyses were conducted using SPSS software (version 23.0; IBM Corp., Armonk, NY, USA) and GraphPad Prism. A p‐value < 0.05 was considered statistically significant.

## Conflict of Interest

The authors declare no conflict of interest.

## Author Contributions

J.T.Z., M.G., and S.K.Z. contributed equally to this work. J.T.Z., M.G., and S.K.Z. performed the experiments, analyzed data, and wrote the manuscript. W.W.G. performed animal experiments. M.H.Z. analyzed the data. W.Z.H. provided technical support. X.M.L., W.J.M., and Y.F.Y. supervised the study.

## Supporting information



Supporting Information

## Data Availability

The data that support the findings of this study are available from the corresponding author upon reasonable request.
