# Supplementary material for: PP1A Modulates the Efficacy of Lenvatinib Plus ICIs Therapy by Inhibiting Ferroptosis in Hepatocellular Carcinoma
Source: Adv Sci (Weinh). 2025 May 8;12(27):2501730. doi: 10.1002/advs.202501730 (PMC12279197; doi:10.1002/advs.202501730)
Supplement: Supplementary file 1 — Supporting Information [file ADVS-12-2501730-s001.docx]

**PP1A Modulates the Efficacy of Lenvatinib Plus ICIs Therapy by Inhibiting Ferroptosis in Hepatocellular Carcinoma**

**Jitong Zhou1,2#, Meng Gao1,2#, Shikun Zhang1,2#, Wing-Wa Guo1,2, Wenzhi He1,2, Minghe Zhang1,2, Xi Chen1,2，Cairang Dongzhi1,2，Xiaomian Li1,2*, Yufeng Yuan1,2,3*, Weijie Ma1,2***

**Supplementary Methods**

**Single-cell dissociation**

Single-cell RNA sequencing was conducted by personnel at CapitalBio Technology. Tissue samples were surgically excised and preserved in MACS Tissue Storage Solution (Miltenyi Biotec, Germany) until further processing. Briefly, the samples were washed with phosphate-buffered saline (PBS), minced into approximately 1mm³ pieces on ice, and subjected to enzymatic digestion with 1 mg/mL collagenase II (Worthington, China) for 30 minutes at 37°C with gentle agitation. Following digestion, the samples were passed through a 70-µm cell strainer and centrifuged at 300 g for 5 minutes. After removing the supernatant, the resulting cell pellet was resuspended in red blood cell lysis buffer (Miltenyi Biotec, Germany) to eliminate red blood cells. The cells were then washed with PBS containing 0.04% BSA, resuspended in the same PBS-BSA solution, and filtered through a 35-µm cell strainer. Viability of the dissociated single cells was assessed by staining with AO/PI and analyzed using a Countstar fluorescence cell analyzer. The single-cell suspension was subsequently further enriched using a MACS Dead Cell Removal Kit (Miltenyi Biotec, Germany).

**RNA isolation and qRT-PCR analysis**

Total RNA was extracted using TRIzol Reagent (Takara, Japan) following the manufacturer's instructions. First-strand cDNA was synthesized using a reverse transcriptase cDNA synthesis kit (Takara, Japan), and qRT-PCR analysis was performed using the SYBR Green PCR Kit (Takara, Japan ). Each qRT-PCR reaction (20 μL) contained 500 ng of cDNA along with specific forward and reverse primers. The sequences of all primers are provided in the Supplementary Table S1. Gene expression levels were quantified using the 2−ΔΔCt method and normalized to GAPDH expression. All experiments were performed in triplicate.

**Immunofluorescence (IF) analyses**

For immunofluorescence (IF) analysis, cells were washed three times with phosphate-buffered saline (PBS) and fixed with 4% paraformaldehyde for 20 minutes. HCC cells were subsequently permeabilized with 0.5% NP-40 in PBS for 20 minutes and blocked with 5% bovine serum albumin (BSA) for 30 minutes. Following the blocking step, cells were incubated with primary antibodies for 2 hours, followed by incubation with secondary antibodies for 1 hour. Nuclei were stained with DAPI (ABclonal, RM02978, China). Images were acquired using a confocal microscope (OLYMPUS, IX51).

**Immunohistochemistry (IHC) analyses**

For immunohistochemistry (IHC), paraffin-embedded sections were baked at 65°C for 2 hours, deparaffinized, and rehydrated. Antigen retrieval was performed using EDTA buffer, followed by incubation with 3% hydrogen peroxide for 10 minutes to block endogenous peroxidase. Sections were blocked with 5% BSA for 20 minutes, then incubated with diluted primary antibody overnight at 4°C. HRP-conjugated secondary antibody was applied, followed by incubation for 50 minutes. DAB solution was used for visualization, and sections were counterstained with hematoxylin, dehydrated, and cleared in xylene. Two pathologists independently scored the IHC sections.

**RNA-sequencing (RNA-seq)**

RNA integrity was assessed using the Agilent 2100 Bioanalyzer, and only samples with an RNA integrity number (RIN) > 7 and appropriate purity (A260/280 between 1.8 and 2.2) were used for library preparation. Libraries were constructed using the VAHTS mRNA-seq v2 Library Prep Kit for Illumina, following the manufacturer's protocol, and sequenced on the Illumina NovaSeq platform with 150 bp paired-end reads for high depth and coverage.Raw data underwent quality control to remove adapter sequences, low-quality reads (Q < 20), and reads with more than 10% ambiguous bases. Clean reads were aligned to the human reference genome using HISAT2, and gene expression was quantified using the FPKM method. Differential expression analysis was performed with DESeq2, defining differentially expressed genes (DEGs) as those with a P-value < 0.05 and an absolute log2 fold change > 1.

**Cell apoptosis analysis**

HCC cells were cultured in six-well plates until they reached 80% confluence, after which the serum-containing medium was replaced with serum-free medium. Following 24 hours of incubation in serum-free conditions, all cells were collected. Apoptosis was assessed using the Annexin V-FITC apoptosis detection kit (Lianke, Hangzhou, China). Briefly, HCC cells were stained with Annexin V-FITC and Propidium Iodide (PI) and analyzed using a Cytoflex flow cytometer (Beckman, China) within 5 minutes of staining. The data were processed using FlowJo software (version 10.6.0, Ashland, OR, USA).

**Cell proliferation assays**

CCK-8 and colony formation assay: Cell proliferation was assessed by CCK-8 assay (MCE, USA). Cells (2×103cells/well) were plated in 96 well plates with 200uL of culture medium and cultured for the indicated time. With supernatant removed, CCK-8 reagent was added to each well and incubated for 1 h at 37℃. The absorbance at 450nm was detected using a microplate reader (Bio-Rad, USA).For colony formation assay, cells (1×103 cells/well) were seeded into 6 well plates and incubated for 2-3 weeks. Then, the colonies on the plates were fixed with 4% polyoxymethylene and stained with 0.1% crystal violet.

**Scratch wound-healing motility assay**

cells (5 × 104) were seeded into both sides of Culture-Inserts® (Ibidi, Germany) to make a 500μm gap. After incubation for 24 h, inserts were removed to allow cell migration for the indicated period of time.

**Eukaryotic protein expression and purification**

Plate 293T cells to reach 80% confluency on the day of transfection. Transfect the cells with a Flag-tagged plasmid and incubate for 48 hours. Wash twice with cold PBS and detach cells. Collect the cells in cold PBS and pellet them at 1,000×g for 5 minutes at 4°C. Resuspend the pellet in about 1 mL of ice-cold lysis buffer, incubate on ice for 1 hour. Clarify the lysate by centrifuging at 12,000×g for 20 minutes. Equilibrate the anti-Flag beads by washing it 3-5 times with lysis buffer. Add the clarified lysate to the equilibrated resin in a suitable tube. Incubate the mixture on a rotator at 4°C overnight. Wash the resin 3-5 times with a 5×resin volume of lysis buffer. Prepare the elution buffer by supplementing lysis buffer with synthetic Flag peptide (MCE, China), then add the elution buffer to the resin (500 µL per purification) and gently agitate at 4°C for 1-2 hours. Collect the supernatant containing the eluted Flag-tagged protein.

**In vitro phosphatase assays**

Proteins obtained in the “Eukaryotic Protein Expression and Purification” section were used in this experiment. Add the substrate (Keap1/Keap1-S104E) and enzyme (PP1A/PP1A-MUT) in an appropriate ratio (approximately 5:1) to about 250 µL of dephosphorylation buffer containing 50 mM HEPES, 100 mM NaCl, 1 mM MnCl2, 2 mM DTT, 0.1 mM EGTA, and 0.025% Tween 20. Incubate the mixture at 37°C for 1.5 hours. Since this experiment tests phosphatase activity, the substrate (Keap1/Keap1-S104E) is ideally pre-phosphorylated using a kinase before the reaction. After incubation, analyze the reaction products by Western blot. A phospho-specific antibody was used to assess the phosphorylation level of the substrate. All antibodies are listed in Supplementary Table S2.

**Transwell invasion assay**

cells (5 × 104) suspended in serum-free medium were seeded into each upper chamber with or without pre-coated Matrigel (Corning, USA), while medium containing 10% FBS was added to the lower chamber. After incubation for 48 h, the migratory or invasive cells in the lower chambers were fixed with 4% paraformaldehyde and stained with crystal violet.

**Flow cytometry**

Cells were obtained after co-culture. After incubated with Fc block, cells were stained with surface or intracellular primary antibodies (Biolegend, USA) according to the manufacturer’s protocols. Stained cells were acquired on a Flow Cytometer (BD FACSCanto II or BD LSRFortessa, USA) and the results were analyzed by FlowJo software. The antibodies used for flow cytometry are listed in **Supplementary Table S2**.

**Western blot**

The samples were lysed in RIPA Buffer (Servicebio, China) supplemented with protease and phosphorylation inhibitor cocktail (Beyotime, China). Equal amounts of protein were separated by SDS-PAGE and transferred to PVDF membranes (Millipore, USA). After blocking with 5% nonfat milk, the membranes were incubated overnight at 4 °C with primary antibodies and then incubated with HRP-conjugated secondary antibodies (1:5000; Abclonal, China) for 1h. Finally, signals were detected with an enhanced chemiluminescence kit and visualized in a ChemiDoc MP Imaging System. Antibodies used in this study are provided in **Supplementary Table S2**.

**Table S1. Primers for quantitative real-time PCR and ChIP-PCR analysis**

| Gene | Primers |
| --- | --- |
| hGAPDH-F | AGGGCTGCTTTTAACTCTGGT |
| hGAPDH-R | CCCCACTTGATTTTGGAGGGA |
| hPP1A-F | AGTACTACGACCTTCTGCGACTA |
| hPP1A-R | ACAGTTTGATGTTGTAGCGTCTC |
| hNrf2-F | CACATCCAGTCAGAAACCAGTGG |
| hNrf2-R | GGAATGTCTGCGCCAAAAGCTG |
| hPD-L1-F | TGCCGACTACAAGCGAATTACTG |
| hPD-L1-R | CTGCTTGTCCAGATGACTTCGG |
| hGPX4-F | ACAAGAACGGCTGCGTGGTGAA |
| hGPX4-R | GCCACACACTTGTGGAGCTAGA |
| hHO-1-F | CCAGGCAGAGAATGCTGAGTTC |
| hHO-1-R | AAGACTGGGCTCTCCTTGTTGC |
| hNQO1-F | CCAGGCAGAGAATGCTGAGTTC |
| hNQO1-R | GTGGTGATGGAAAGCACTGCCT |
| hPD-L1-WT1-F | GGGCTTTCTTAACCCTCACC |
| hPD-L1-WT1-R | GACCCATATGGCTTTGGTTTT |
| hSLC3A2-F | GCCAGGACACCGAGGTG |
| hSLC3A2-R | CCTCCGCCTCGTCTTCCG |
| mGAPDH-F | ATGCCAGTGAGCTTCCCGTTCAG |
| mGAPDH-R | GAGCCTACTTTGTACGTGGCGA |
| mPP1A-F | AGCGAGAAGCTCAACCTGGA |
| mPP1A-R | ACCTCAGCCCCAAAGGTAAAG |
| hKeap1-F | GCCGATTCCTGCCCCTG |
| hKeap1-R | AGTTGGCAGTGGGACAG |

**Table S2. Antibodies used in this study**

| Antibodies | Company | Cat no. | Clone no. |
| --- | --- | --- | --- |
| GAPDH | Proteintech | 60004-1-Ig | 1E6D9 |
| PP1A | Proteintech | 67070-1-Ig | 1C10A2 |
| PP1A | Abclonal | A24288 | ARC61729 |
| Nrf2 | Proteintech | 80593-1-RR | 1I21 |
| GPX4 | Proteintech | 67763-1-Ig | 3F5G5 |
| Nrf2 | Abcam | ab137550 |  |
| KEAP1 | Proteintech | 60027-1-Ig | 4G10H9 |
| KEAP1 | Abclonal | A25297 | ARC3231 |
| NQO1 | Abclonal | A23486 | ARC0065 |
| NQO1 | Proteintech | 67240-1-Ig | 1E5G7 |
| Ki67 | Abclonal | A21861 | ARC5050-01 |
| FLAG | Proteintech | 20543-1-AP | 1010 |
| HA | Proteintech | 51064-2-AP | 825 |
| Anti-Phospho-(Ser/Thr) | Abcam | ab300625 |  |
| HO-1 | Proteintech | 66743-1-Ig | 2D10A5 |
| PD-L1 | Proteintech | 66248-1-Ig | 2B11D11 |
| CD4 | Proteintech | APC-98042 | 240427E12 |
| CD8 | Proteintech | 65559-1-MR | UCHT4 |
| ACSL4 | Proteintech | 66617-1-Ig | 1H5D3 |
| PD-1 | BioXCell | BE0146 | RMP1-14 |
| IgG2aisotype | BioXCell | BE0089 | 2A3 |
| PD-L1 | Biolegend | 329706 | 29E.2A3 |
| IgG2bisotype | Biolegend | 400314 | MPC-11 |
| PD-L1 | Biolegend | 124311 | 10F.9G2 |
| IgG2bisotype | Biolegend | 400611 | RTK4530 |
| CD45,PE | Biolegend | 147712 | I3/2.3 |
| CD3,APC | Biolegend | 100236 | 17A2 |
| CD3,BV421 | Biolegend | 100228 | 17A2 |
| CD4,PE/Cyanine7 | Biolegend | 100422 | GK1.5 |
| CD4,BV605 | Biolegend | 100451 | GK1.5 |
| CD8a,FITC | Biolegend | 100706 | 53-6.7 |
| CD8a,AF700 | Biolegend | 100729 | 53-6.7 |
| GranzymeB,PE/Cyanine7 | Biolegend | 372214 | QA16A02 |
| TNF-α,PE | Biolegend | 506306 | MP6-XT22 |

**Table S3. Sequences of shRNAs and siRNAs in this study**

| Name | Sequences |
| --- | --- |
| h-siPP1A-1 | Sense CUACGACCUUCUGCGACUATT |
| h-siPP1A-1 | Anti-Sense UAGUCGCAGAAGGUCGUAGTT |
| h-siPP1A-2 | Sense GAGACGCUACAACAUCAAATT |
| h-siPP1A-2 | Anti-Sense UUUGAUGUUGUAGCGUCUCTT |
| h-siPP1A-3 | Sense CUGGCCUAUAAGAUCAAGUTT |
| h-siPP1A-3 | Anti-Sense ACUUGAUCUUAUAGGCCAGTT |
| h-sh-PP1A-1 | ACTACGACCTTCTGCGACTAT |
| h-sh-PP1A-2 | GAGACCCTCATGTGCTCTTTC |
| h-sh-PP1A-3 | TGAGTGCAAGAGACGCTACAA |
| m-sh-PP1A-1 | CGTGGTGTCTCCTTTACCTTT |
| m-sh-PP1A-2 | CCGGAGAATTTCTTTCTACTT |
| m-sh-PP1A-3 | CGCTGATAAGAATAAGGGCAA |

**Table S4. Bliss independence model used to assess the potential synergistic effect**

| vector+DMSO | vector+Lenvatinib | shPP1A+DMSO | shPP1A+Lenvatinib |
| --- | --- | --- | --- |
| 1.712 | 1.397 | 1.418 | 1.197 |
| 1.659 | 1.351 | 1.351 | 1.260 |
| 1.764 | 1.333 | 1.544 | 0.840 |
| 1.806 | 1.502 | 1.565 | 1.302 |
| 1.680 | 1.473 | 1.368 | 1.061 |

Bliss Independence Model

(1) Calculation of Bliss Predicted Effect:

E^Bliss^_AB_ = E_A_ + E_B_ - (E_A_ × E_B_)

(2) Calculation of Synergistic Effect (ΔE):

ΔE = E^actual^_AB_ − E^Bliss^_AB_

ΔE > 0: Synergistic effect

ΔE = 0: Independent effect

ΔE < 0: Antagonistic effect）

In Figure 7O and Figure S8G, the experimental group data related to Lenvatinib and ShPP1A are as follows: (/cm^3^)

Considering "vector + DMSO" as the control group, the tumor inhibition rate for other groups is calculated as:

E = (X_ctrl_ - X)/ X_ctrl_

where:

X_ctrl_ represents the tumor size in the control group

X represents the tumor size in the experimental group

E denotes the inhibition rate

The calculation process is as follows:

E_Lenvatinib_ = 0.181546894031669

E_Sh-PP1A_ = 0.159622411693057

E^actual^_（Lenvatinib + Sh-PP1A）_= 0.343483556638246

E^Bliss^_（Lenvatinib + Sh-PP1A）_ = E_Lenvatinib_ × E_Sh-PP1A_ = 0.312190353

ΔE = E^actual^_（Lenvatinib + Sh-PP1A）_ − E^Bliss^_（Lenvatinib + Sh-PP1A）_= 0.031293204 > 0

Therefore, there is a synergistic effect when Lenvatinib and ShPP1A are combined for HCC treatment.

**Supplementary Figures and Figure legends**

**
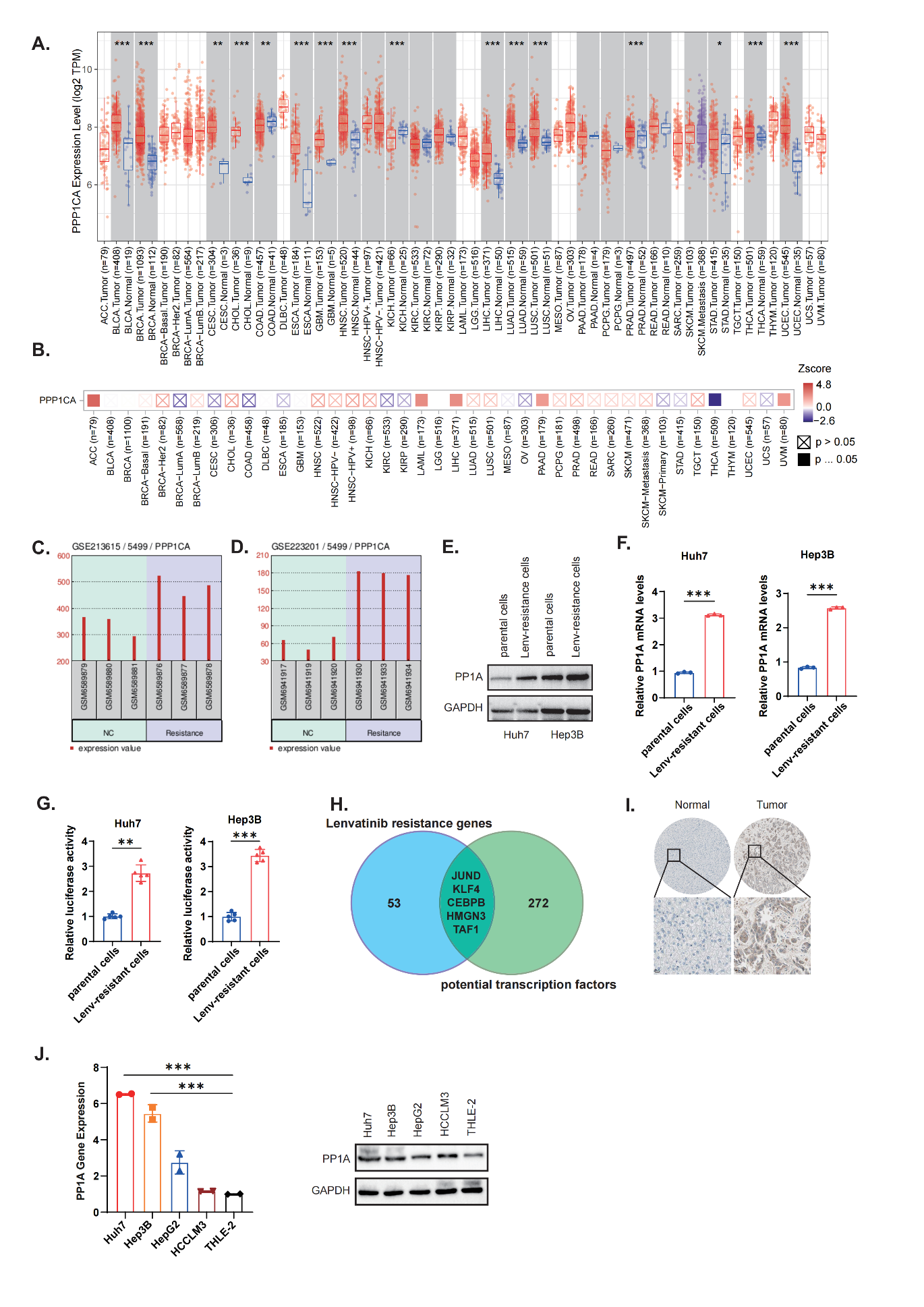
**

**Supplementary Figure 1. PP1A upregulation in HCC predicts poor prognosis and drives resistance to Lenvatinib**

A-B) The HPA (Human Protein Atlas) database represent different expression of PP1A in various tumor cells and its association with prognosis. C-D) The GEO microarray data represents the expression of PP1A in Sorafenib/Lenvatinib-resistant cell lines or patient tissues. E) Western blot detected the expression of PP1A in Lenvatinib-resistant cells and parental cells. F) RT-qPCR detected the mRNA levels of PP1A in Lenvatinib-resistant cells and parental cells (n = 3). G) Dual-luciferase reporter assays were performed to assess the transcriptional activity of PP1A in Lenvatinib-resistant and parental cells (n = 3). H) Venn diagram of transcription factors (JUND, KLF4, CEBPB, HMGN3) associated with both Lenvatinib resistance and PP1A regulation. Data sources include CHEA TFT Binding Site, Cistrome DB, GeneCards, and ENCODE TFT. I) Representative IHC images of PP1A expression from the HPA database. J) Comparison of PP1A expression levels between various liver cancer cell lines and normal liver cell lines using RT-qPCR and Western blot. Unpaired Student's t-test and one-way ANOVA was used to analyze the data. *p < 0.05, **p < 0.01, ***p < 0.001. Data are expressed as mean ± SD from three independent experiments.


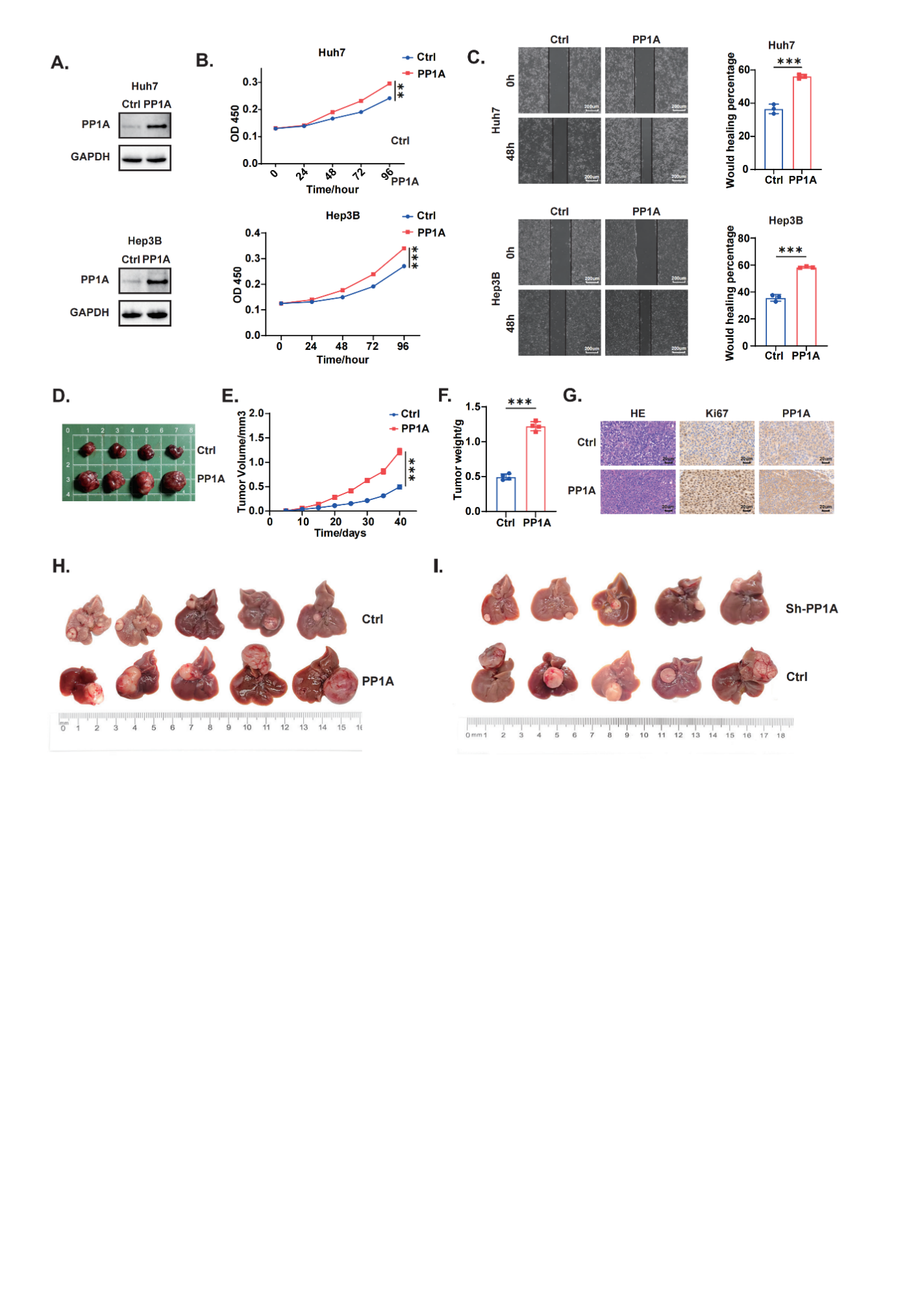


**Supplementary Figure 2.** **PP1A enhances proliferation, invasion, and metastasis of HCC cells in vitro and in vivo**

A) Validation of PP1A expression following overexpression in the indicated cell lines was conducted using Western blot. B) The proliferation of Huh7 and Hep3B cells was detected by CCK-8 assays. C) The migratory capabilities of Huh7 and Hep3B cells were evaluated using wound healing assay (scale bar, 200 μm). D-F) Images of subcutaneous xenograft tumors in nude mice and statistical analyses of tumor volumes and tumor weights in different groups. (n = 5, data missing: one mouse from the Ctrl group and one mouse from the PP1A group died during the experiment.) G) Representative images of IHC staining of PP1A, and Ki67 in subcutaneous xenografts tumor tissues. H) In situ liver cancer models of mice: control and PP1A overexpression groups. I) In situ liver cancer models of mice: control and PP1A knockdown groups. Unpaired Student's t-test was used to analyze the data. *p < 0.05, **p < 0.01, ***p < 0.001. Data are expressed as mean ± SD from three independent experiments.


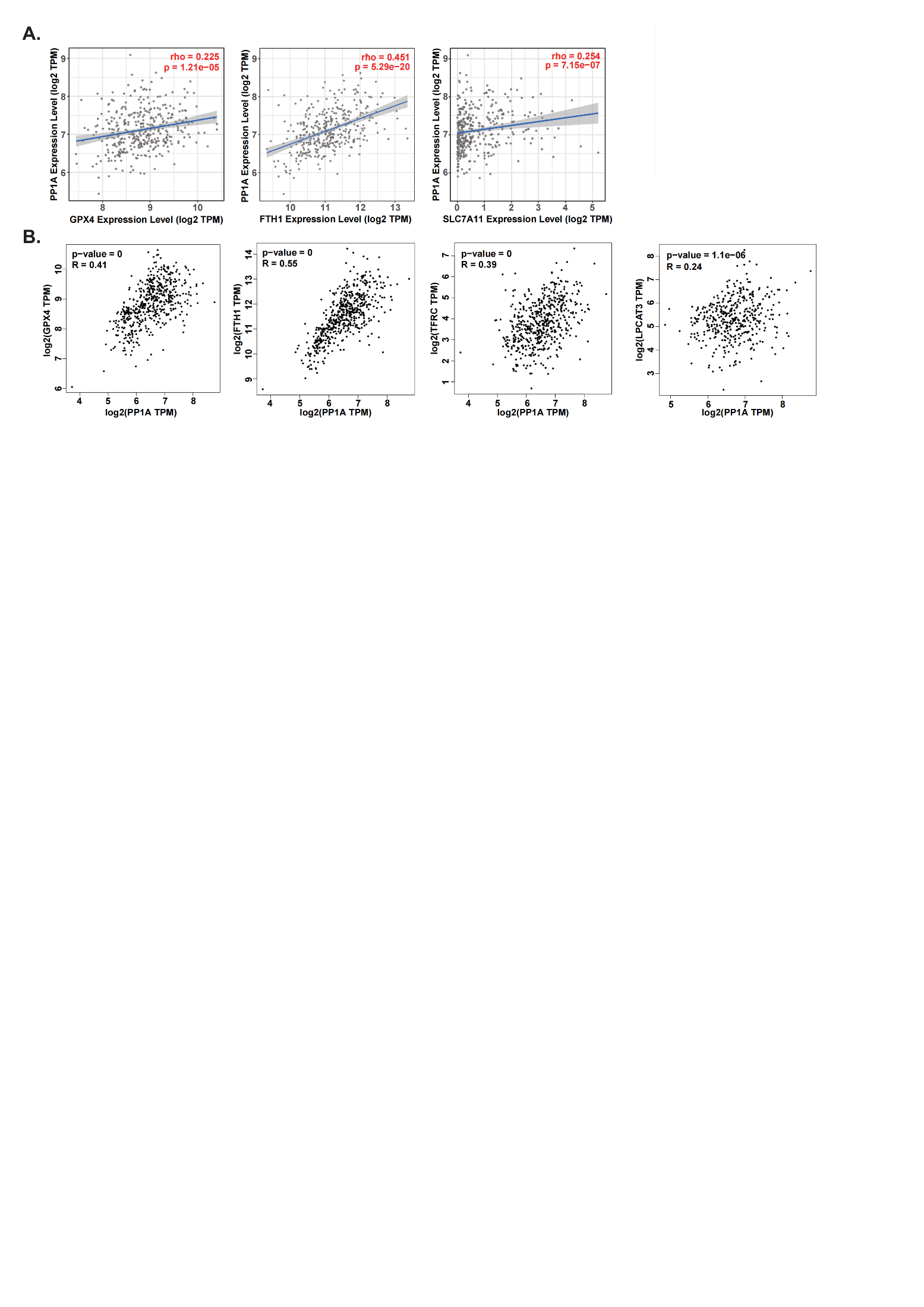


**Supplementary Figure 3. PP1A inhibits Lenvatinib-induced ferroptosis in HCC**

A) The TIMER2.0 database was used to analyze the correlation between PP1A and GPX4, SLC7A11, and FTH1. B) Gepia2 was used to analyze the correlation between PP1A and GPX4, FTH1, TFRC, and LPCAT3. Pearson correlation analysis was used to analyze the data.


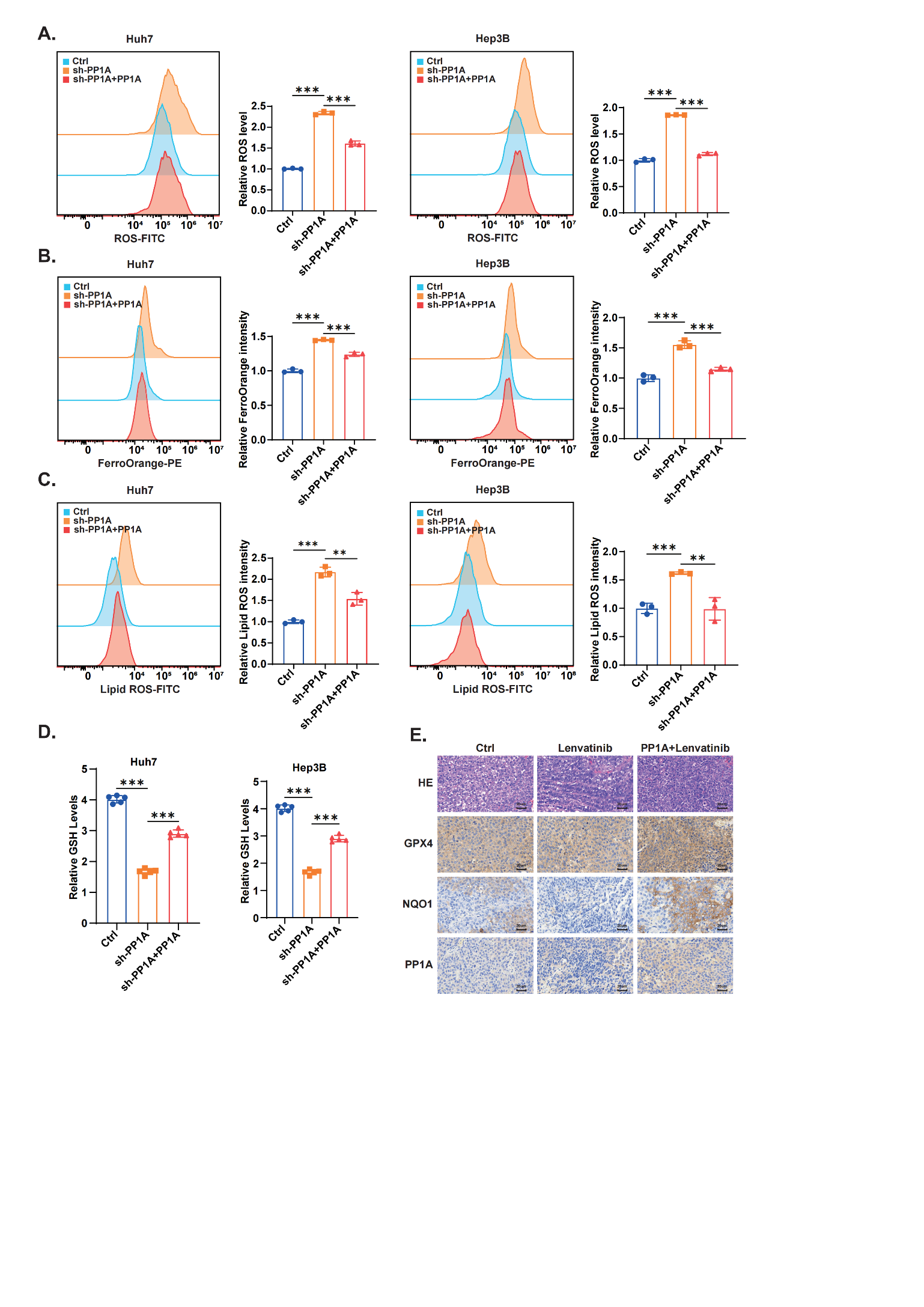


**Supplementary Figure 4. PP1A inhibits Lenvatinib-induced ferroptosis in HCC**

A) Intracellular ROS levels in Huh7 and Hep3B cells were detected by flow cytometry using DCFH-DA. B) Intracellular Fe²⁺ levels in Huh7 and Hep3B cells were measured by flow cytometry using FerroOrange. C) Lipid ROS accumulation in Huh7 and Hep3B cells was assessed by flow cytometry using C11-BODIPY 581/591. D) Intracellular GSH levels in Huh7 and Hep3B cells were quantified using a GSSG/GSH quantification kit. E) Representative images of IHC staining of GPX4, NQO1 and PP1A in subcutaneous xenografts tumor tissues. Unpaired Student's t-test or one-way ANOVA was used to analyze the data. *p < 0.05, **p < 0.01, ***p < 0.001. Data are expressed as mean ± SD from three independent experiments.

**
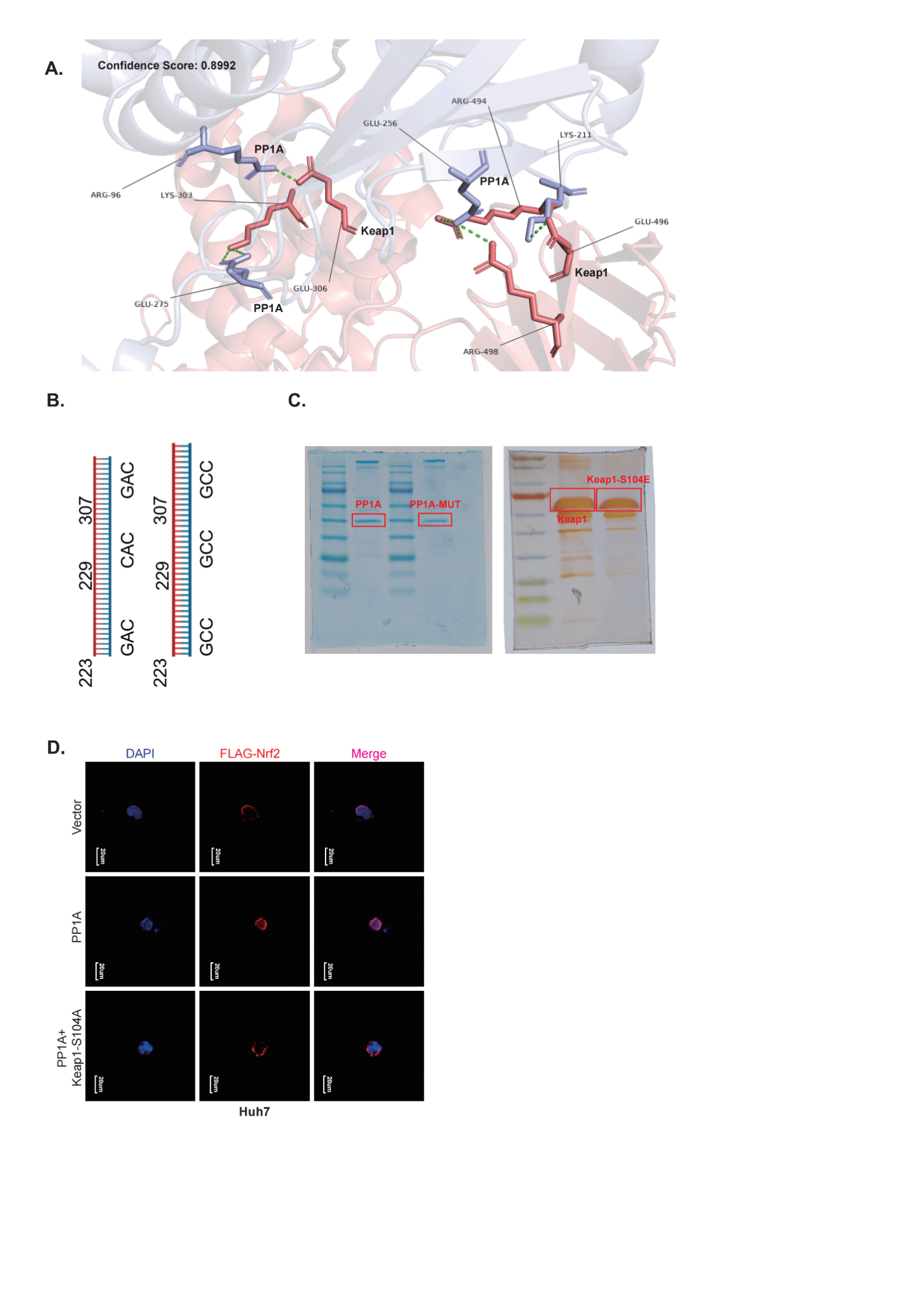
**

**Supplementary Figure 5. PP1A-mediated dephosphorylation of KEAP1 stabilizes Nrf2 protein levels by decreasing interaction of KEAP1 and Nrf2**

A) Molecular docking was used to assess the interacion of PP1A and KEAP1. B) Schematic diagram of mutation treatment at the phosphatase active site of PP1A. C) Coomassie Brilliant Blue staining/Silver staining of purified PP1A, Keap1, and related mutants. D) Immunofluorescence was used to detect the localization of Nrf2 in the nucleus (scale bar, 20 μm).

**
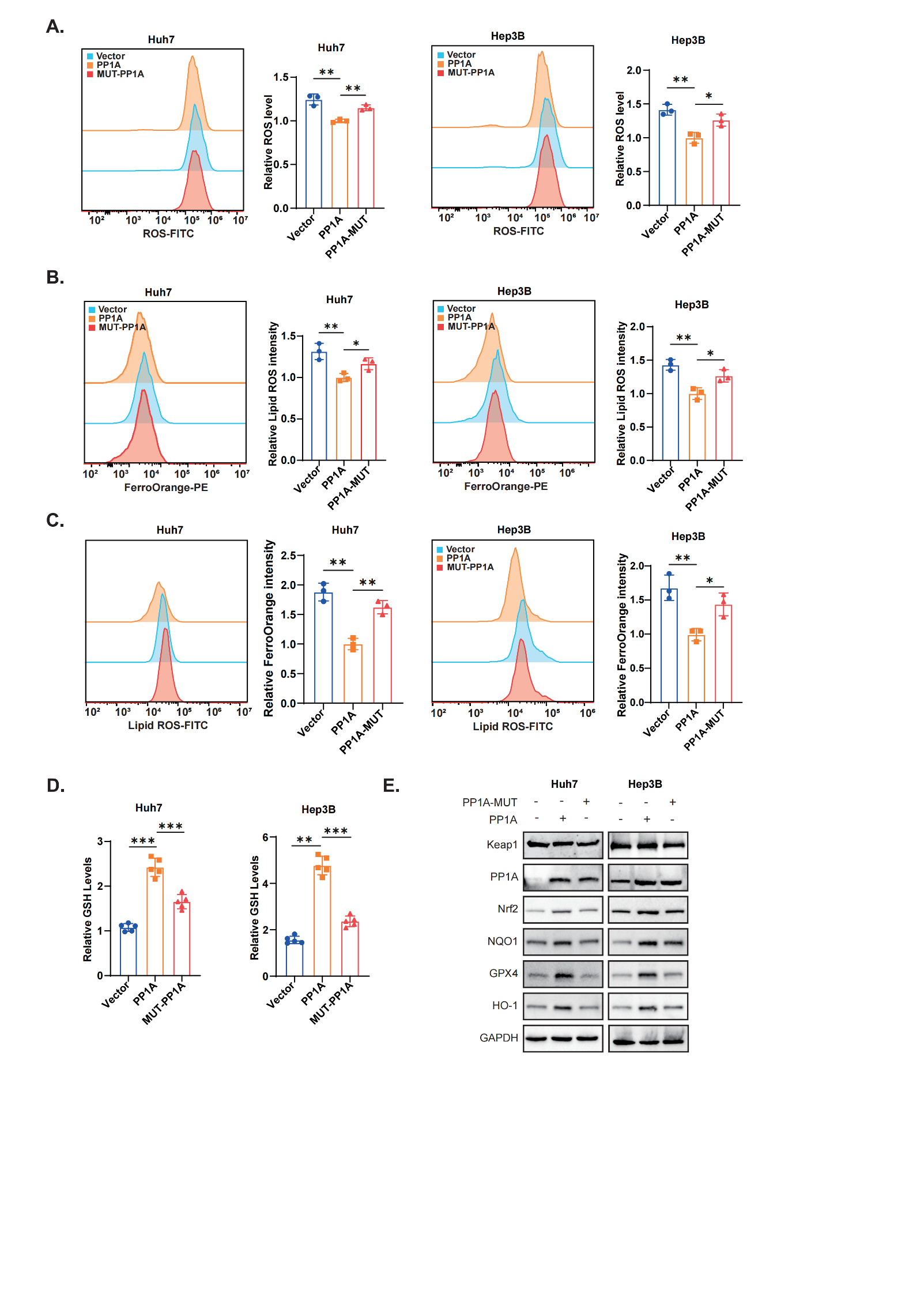
**

**Supplementary Figure 6. Mutation of KEAP1 and PP1A reverses ferroptosis induced by PP1A overexpression**

A) Intracellular ROS levels in Huh7 and Hep3B cells were detected by flow cytometry using DCFH-DA. B) Intracellular Fe²⁺ levels in Huh7 and Hep3B cells were measured by flow cytometry using FerroOrange. C) Lipid ROS accumulation in Huh7 and Hep3B cells was assessed by flow cytometry using C11-BODIPY 581/591. D) Intracellular GSH levels in Huh7 and Hep3B cells were quantified using a GSSG/GSH quantification kit. E) Western blot analysis was performed to detect the expression of ferroptosis-related downstream proteins. Unpaired Student's t-test or one-way ANOVA was used to analyze the data. *p < 0.05, **p < 0.01, ***p < 0.001. Data are expressed as mean ± SD from three independent experiments.


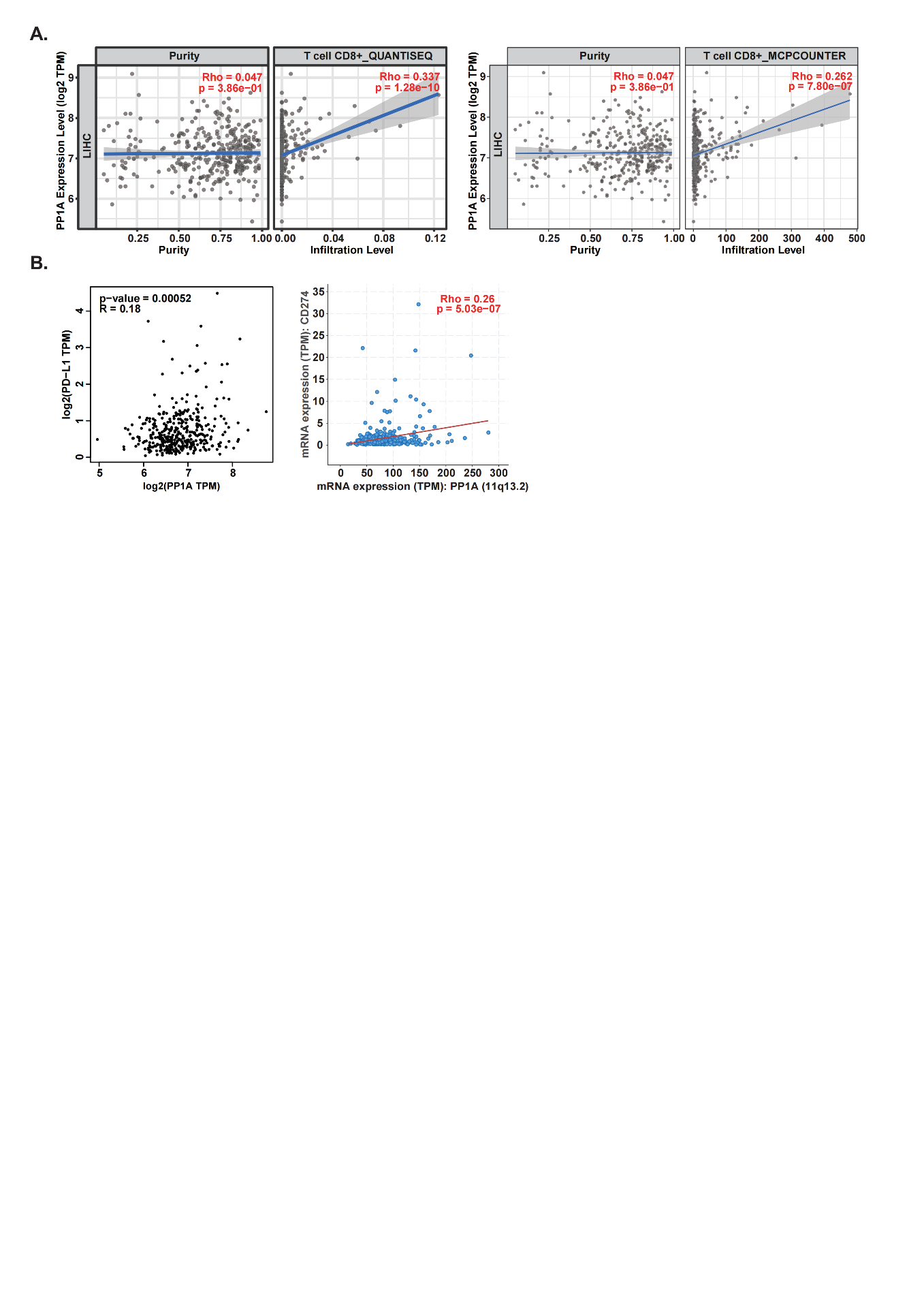


**Supplementary Figure 7. PP1A enhances tumor immunity by upregulating PD-L1**

A) The association between PP1A and the infiltration level of CD8^+^ T cells in the TIMER2.0 database. B) Gepia2 and cBioPortal indicate the correlation between PP1A and PD-L1. Pearson correlation analysis was used to analyze the data.


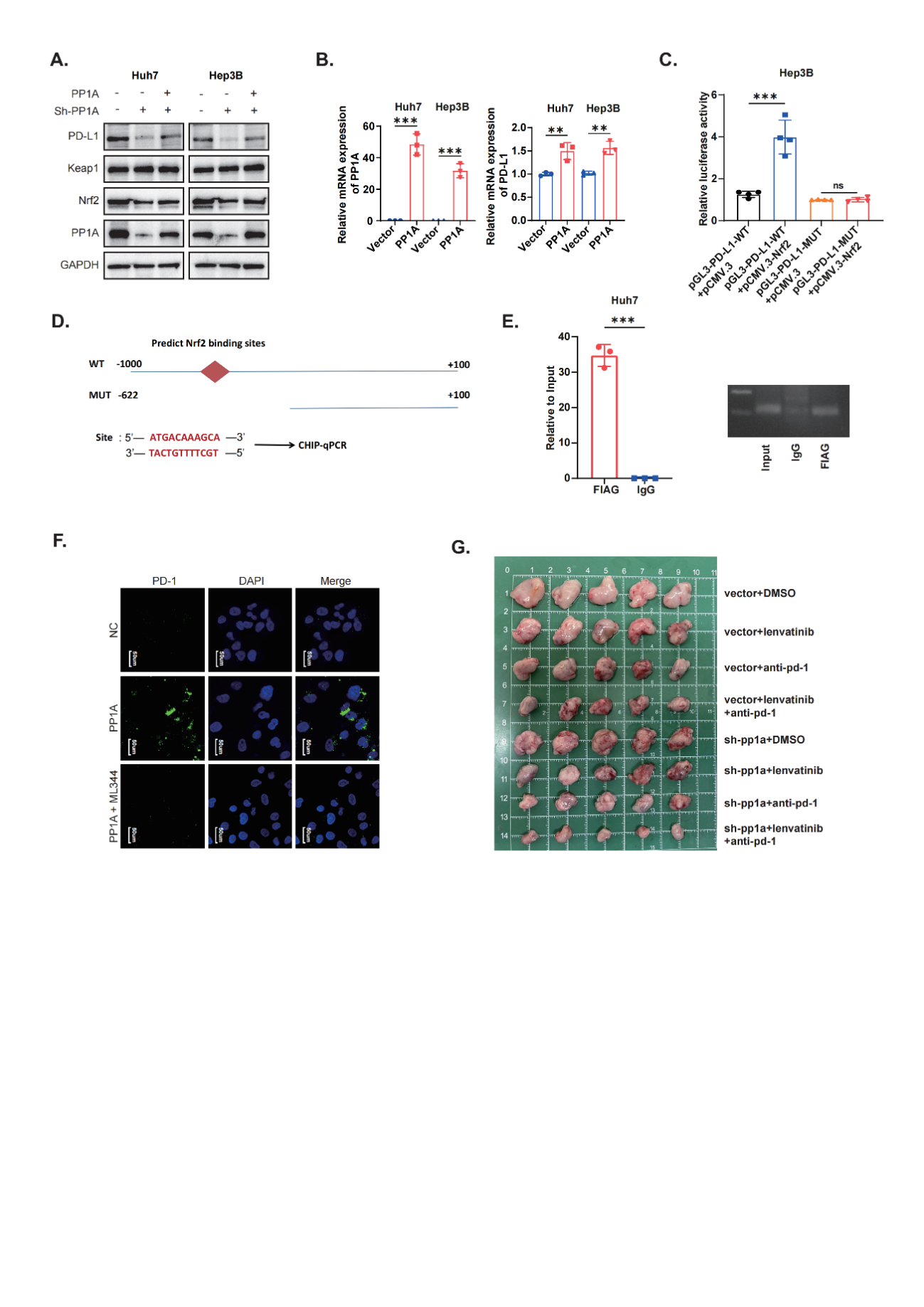


**Supplementary Figure 8.** **PP1A enhances tumor immunity by upregulating PD-L1 and affects Lenvatinib plus immune checkpoint inhibitors efficacy**

A) Western blot analysis of PD-L1 expression changes upon PP1A knockdown and reintroduction of PP1A after knockdown. B) qRT-PCR analysis to investigate the relationship between PP1A and PD-L1 mRNA levels. C) Dual-luciferase reporter assays to assess Nrf2 regulation of PD-L1 transcription levels. D) Schematic diagram of chromatin immunoprecipitation (ChIP) E) Perform PCR amplification of ChIP results and conduct agarose gel electrophoresis. F) Immunofluorescence was used to indirectly assess PD-L1 expression by detecting PD-1 bound to the cell surface. (scale bar, 50μm). G) Representative images of syngeneic tumors in C57BL/6 mice. Unpaired Student's t-test or one-way ANOVA was used to analyze the data. *p < 0.05, **p < 0.01, ***p < 0.001. Data are expressed as mean ± SD from three independent experiments.


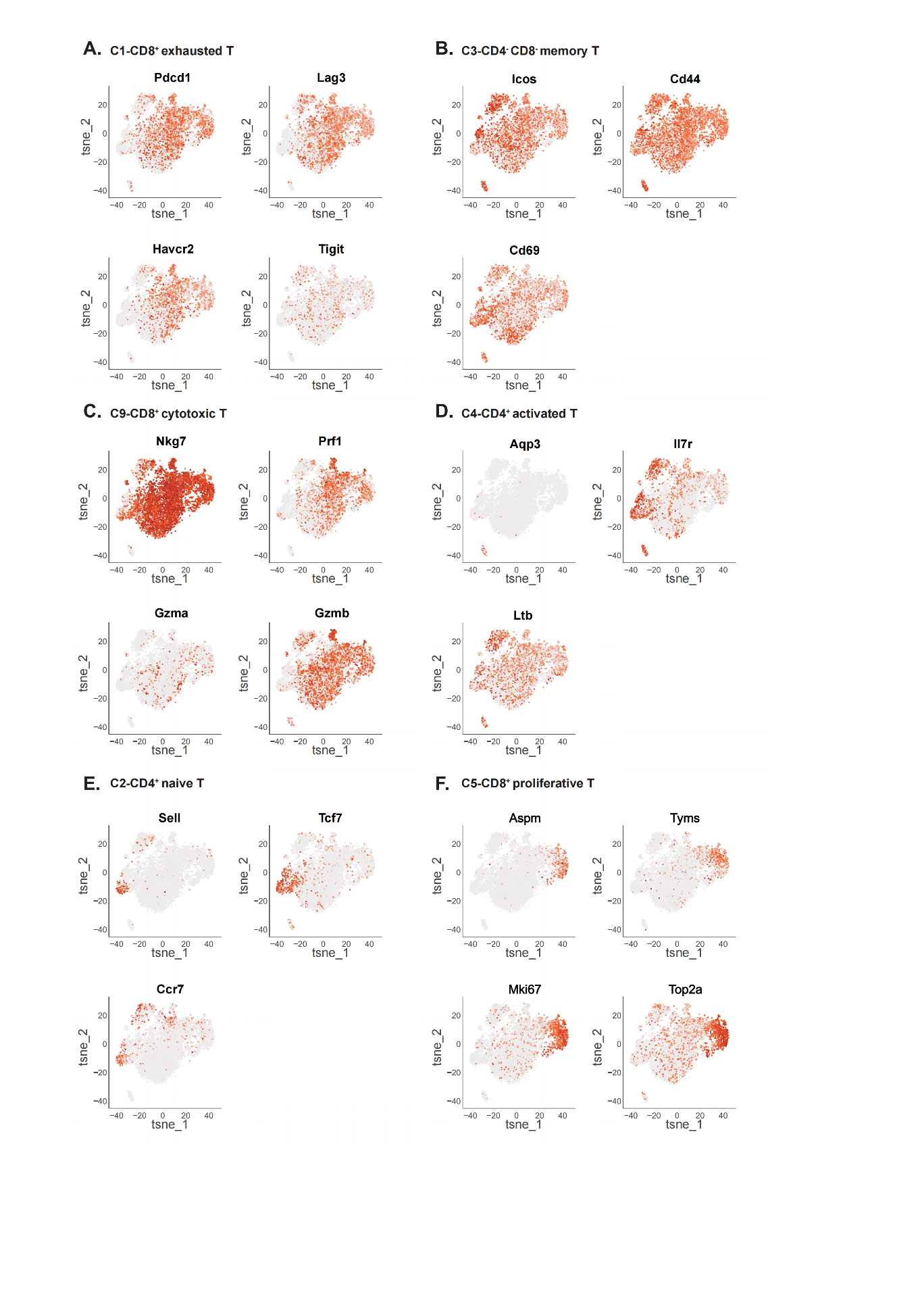


**Supplementary Figure 9. PP1A enhances tumor immunity by upregulating PD-L1 and affects Lenvatinib plus immune checkpoint inhibitors efficacy**

A-F) T cell subset markers and corresponding t-SNE plots.


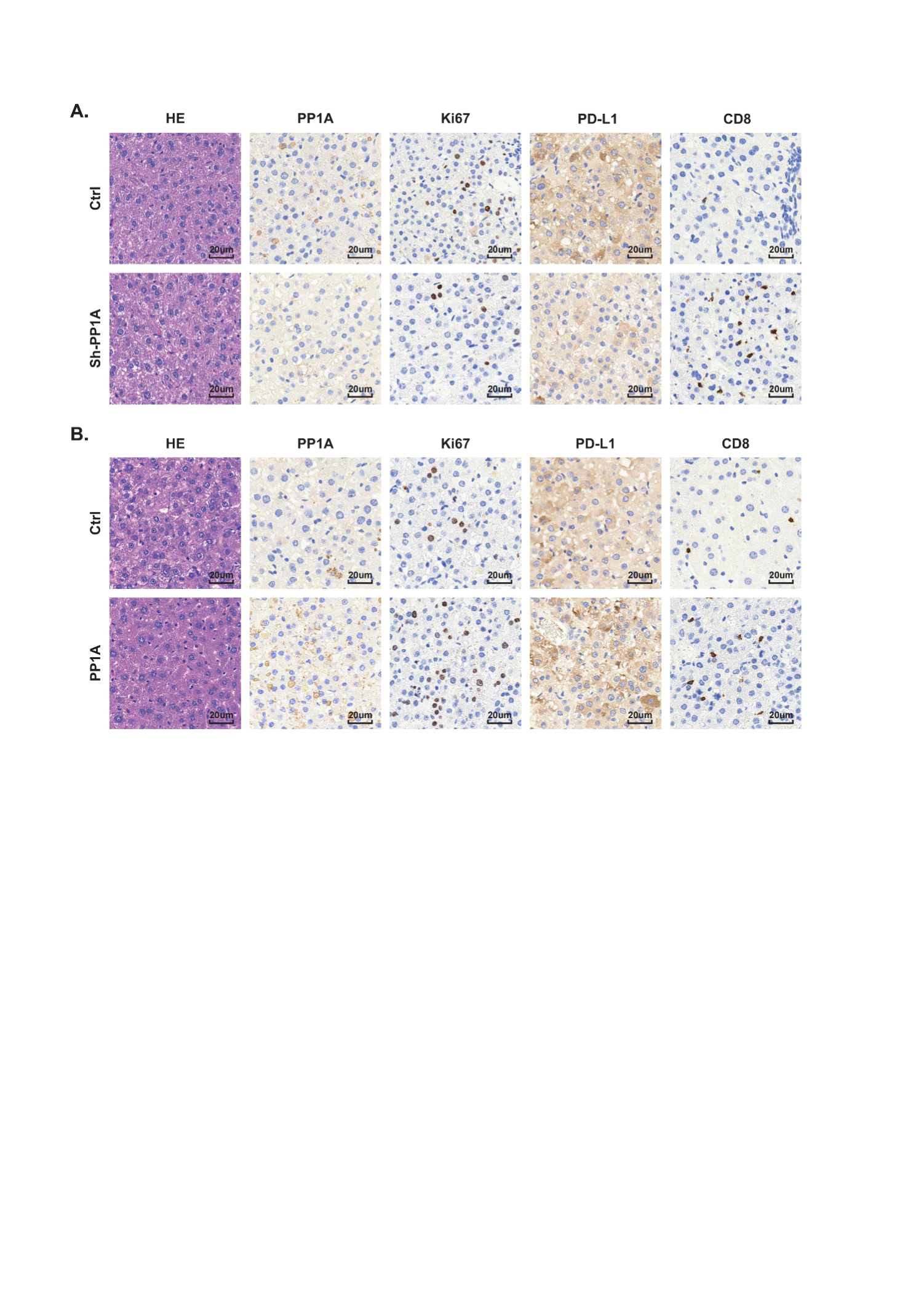


**Supplementary Figure 10. PP1A enhances tumor immunity by upregulating PD-L1 and affects Lenvatinib plus immune checkpoint inhibitors efficacy**

A-B) Representative IHC staining images of PP1A, Ki67, PD-L1, and CD8 in the mouse orthotopic liver cancer model from **Figure S2**.


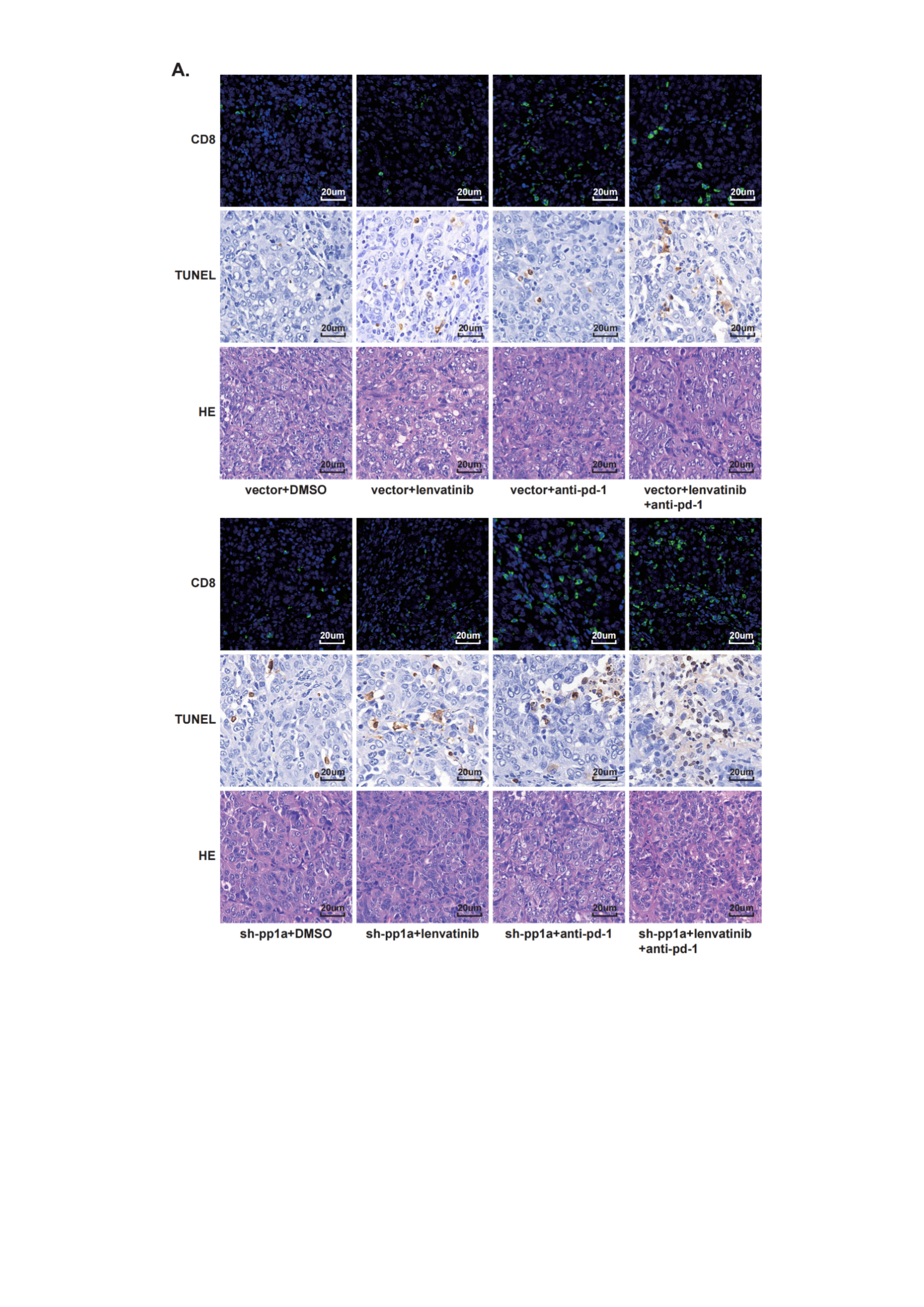


**Supplementary Figure 11.** **PP1A enhances tumor immunity by upregulating PD-L1 and affects Lenvatinib plus immune checkpoint inhibitors efficacy**

A) Representative images of IHC staining for TUNEL and IF staining for CD8 in syngeneic tumor tissues.


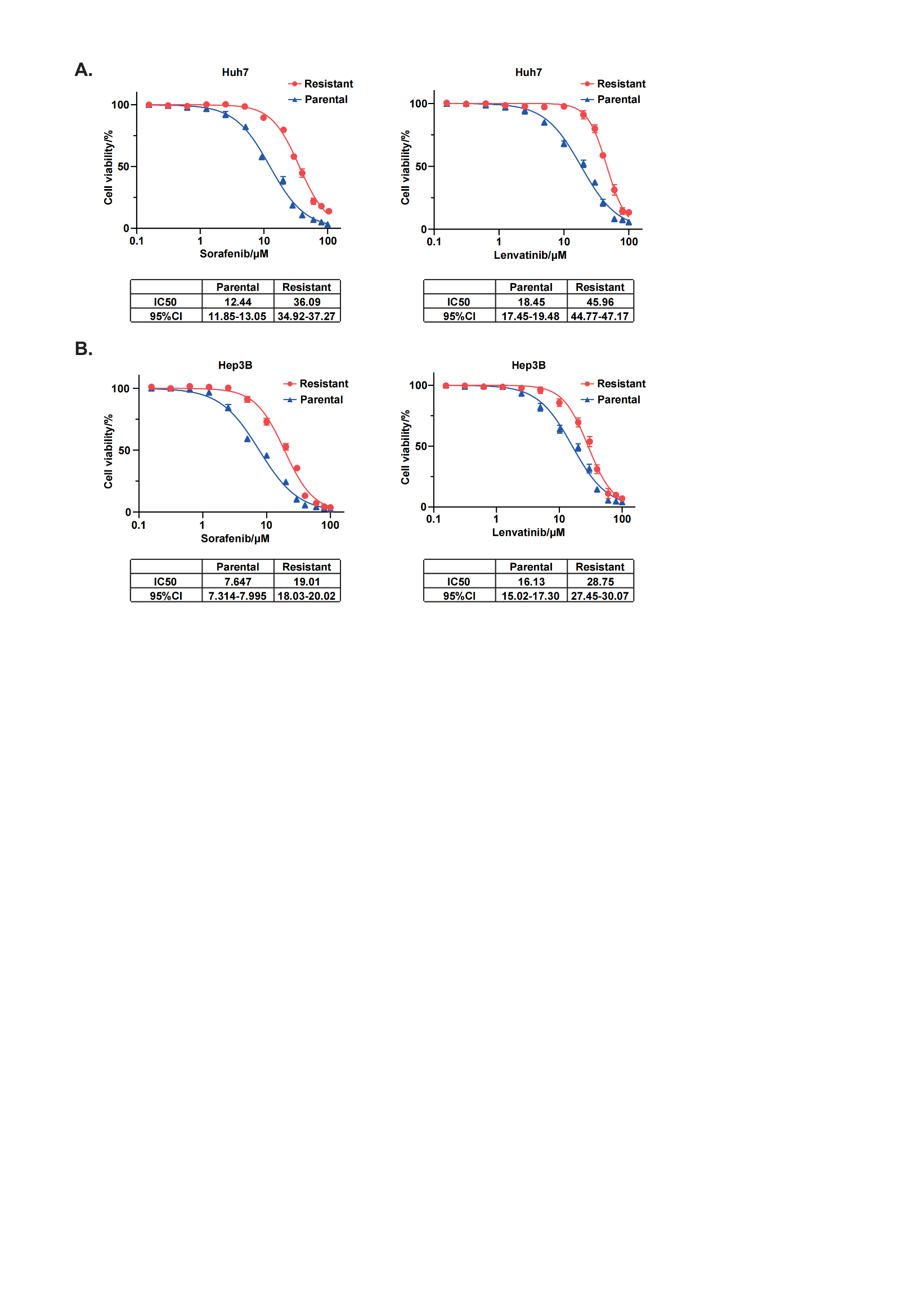


**Supplementary Figure 12.**

A) IC50 of parental Huh7 cell lines and Sorafenib/Lenvatinib-resistant Huh7 cell lines. B) IC50 of parental Hep3B cell lines and Sorafenib/Lenvatinib-resistant Huh7 cell lines. Nonlinear regression was used to analyze the data.
